# Aldehyde dehydrogenase 2 alleviates mitochondrial dysfunction by promoting PGC-1α-mediated biogenesis in acute kidney injury

**DOI:** 10.1038/s41419-023-05557-x

**Published:** 2023-01-20

**Authors:** Jiaying Li, Xiaoxiao Shi, Zhixin Chen, Jiatong Xu, Ruohuan Zhao, Yuhao Liu, Yubing Wen, Limeng Chen

**Affiliations:** 1 Department of Nephrology, State Key Laboratory of Complex Severe and Rare Diseases, Peking Union Medical College Hospital, Chinese Academy of Medical Science and Peking Union Medical College, 100730 Beijing, China; 2 Emergency Department, State Key Laboratory of Complex Severe and Rare Diseases, Peking Union Medical College Hospital, Chinese Academy of Medical Science and Peking Union Medical College, 100730 Beijing, China

**Keywords:** Pathogenesis, Diseases

## Abstract

Renal tubular epithelial cells are one of the high energy-consuming cell types, which mainly depend on mitochondrial energy supply. Aldehyde dehydrogenase 2 (ALDH2) is a key enzyme that is involved in alcohol metabolism and mitochondrial oxidative ATP production; however, its function in mitochondrial homoeostasis in acute kidney injury (AKI) is unclear. Here, we found that ALDH2 expression was predominantly decreased in cisplatin or maleic acid (MA) models both in vivo and in vitro. *ALDH2* knockout (KO) mice exhibited exacerbated kidney impairment and apoptosis of tubular epithelial cells after cisplatin injection. In contrast, ALDH2 activation alleviated AKI and tubular cell apoptosis in both cisplatin- and MA-induced models. RNA sequencing revealed that the oxidative phosphorylation pathway was positively enriched in the renal tissues after Alda-1 pre-treatment in MA-induced mice. ALDH2 activation restored mitochondrial structure, mitochondrial membrane potential, and respiration rate, but downregulated glycolysis in MA-induced mice and human renal proximal tubular epithelial (HK-2) cells. Mechanistically, co-immunoprecipitation assays revealed that ALDH2 interacts with peroxisomal proliferator-γ coactivator-1α (PGC-1α), a master regulator of mitochondrial biogenesis, and advanced its nuclear translocation. Subsequently, *PGC-1α* knockdown almost abolished the improvement of ALDH2 activation on MA-induced tubular epithelial cells damage. Thus, our study revealed that ALDH2 activation alleviated mitochondrial dysfunction in AKI by enhancing PGC-1α-mediated mitochondrial biogenesis. Hence, ALDH2 may act as a potential therapeutic target to prevent AKI progression.

## Introduction

Acute kidney injury (AKI), which is characterised by a rapid decline in renal function, contributes to approximately 1.7 million deaths worldwide each year [1]. Cisplatin is known to induce AKI, restricting the use of platinum-based chemotherapeutics in various cancers because of proximal tubular epithelial cells (PTCs) dysfunction or programmed death [2]. PTCs, which are responsible for 90% of tubular reabsorption, are rich in mitochondria to provide sufficient ATP for active transport. Disorders of mitochondria contribute to transporter dysfunction, which is a known cause of Fanconi syndrome (FS) and AKI. Maleic acid (MA), a cis-isomer of fumarate, induces renal tubular injury by inhibiting Na^+^-K^+^-ATPases and disturbing mitochondrial function and fatty acid metabolism [3–6].

PTCs have high levels of peroxisomal proliferator-γ coactivator-1α (PGC-1α) to fulfil their metabolic energy demands. Mitochondrial dysfunction, characterised by the decreased number and damaged morphology of mitochondria, contributes to AKI [7]. Injured mitochondria cannot produce sufficient ATP to maintain fluid and electrolyte homoeostasis in PTCs. Consequently, adaptation to glycolysis is the dominant metabolic pattern for AKI recovery. Glycolysis results in the conversion of glucose to lactate, which induces fibroblast activation and proliferation in septic AKI and aggravates interstitial fibrosis in the unilateral ureteral obstruction model [8–11]. Therefore, targets for the prevention of mitochondrial dysfunction in AKI are urgently required.

Aldehyde dehydrogenase 2 (ALDH2) is a mitochondrial enzyme that metabolises acetaldehyde to nontoxic acetic acid [12]. The wild-type SNP allele (ALDH2-rs671) is found in 30–50% of East Asians and drastically reduces ALDH2 activity [13]. ALDH2 plays a crucial role in myocardial diseases, pulmonary hypertension, and liver diseases by inhibiting oxidative stress and inflammation, improving mitochondrial function, and regulating autophagy [14–20]. However, few studies have focused on AKI and those have provided inconsistent conclusions. ALDH2 protects against contrast-induced AKI, renal ischaemia-reperfusion injury (IRI), and septic AKI by regulating autophagy and the AKT-mTOR pathway [21–23]. In contrast, continuous infusion of Alda-1, an ALDH2 agonist, exacerbated renal tubular injury in an IRI model, with crystal deposition in the tubules [24].

In this study, we identified an innovative mechanism by which ALDH2 mitigated mitochondrial dysfunction through its interaction with PGC-1α (a transcriptional regulator of mitochondrial genes), thereby regulating mitochondrial biogenesis in AKI. Therefore, ALDH2 may be considered a therapeutic target to prevent AKI.

## Methods

### Animal models

*ALDH2* knockout (KO) mice (Cyagen Biosciences, Santa Clara, CA, USA; genotyping data in Supplementary Fig. S1) and wild male C57BL/6 mice (6–8 weeks, Beijing Vital River Laboratory Animal Technology Company) were kept in the animal centre of Peking Union Medical College Hospital (PUMCH) in a temperature-controlled room (22 °C) in a 12 h light/dark cycle and adaptively fed for one week before the establishment of AKI models. All the animals were randomly assigned into different groups (*n* = 6) without blinding. All animal experiments were approved by the PUMCH Institutional Ethics Committee of Animal Care and Use and conducted in accordance with the National Institutes of Health Guide for the Care and Use of Laboratory Animals.

For cisplatin-induced AKI, mice were intraperitoneally (i.p.) injected with cisplatin (18 mg/kg, single injection) and sacrificed for blood and kidney collection after 72 h. For MA-induced AKI with FS, we injected mice with pH-adjusted to 7.0 MA (1.5 mmol/kg, i.p., single injection) (Sigma Aldrich, St. Louis, MO, USA), collected 24 h urine in MMC100 metabolic cages (Hatteras Instruments, Cary, NC, USA), and harvested blood and kidneys after sacrificing them on days 1 and 7. Furthermore, the ALDH2 agonist (Alda-1, 20 mg/kg) (MCE, Shanghai, China) was i.p. injected 3 d before cisplatin or MA injection.

### Cell culture and treatment

The human renal tubular duct epithelial cells (HK-2) were purchased from the Procell Life Science & Technology Company (CL-0109; Wuhan, China). HK-2 was cultured in DMEM-F12 medium (Gibco, Waltham, MA, USA) with 10% foetal bovine serum (FBS; Gibco), 1% penicillin (100 U/mL), and streptomycin (100 µg/mL) at 37 °C in a 5% CO_2_ environment. Cell identification was confirmed by immunofluorescence staining for E-cadherin, cytokeratin 18, and megalin (Figure S3). The cells were incubated with 1 mM MA (Sigma Aldrich) for 24 h with or without Alda-1 (20 μM) (MCE).

To establish stable *ALDH2*-overexpressing cell lines, *ALDH2* overexpression lentiviruses with green fluorescent protein and a fusion Flag-tagged protein were constructed by GenePharma (Shanghai, China). HK-2 cells were selected using 1 µg/mL puromycin (Solarbio, Beijing, China).

Small interfering RNA (siRNA) targeting PGC-1α (siPGC-1α) was purchased from RiboBio (Guangzhou, China). Short hairpin RNA against ALDH2 (shALDH2) or negative control shRNA (shNC) were purchased from GenePharma. HK-2 cells were transfected using LipofectamineR™ 3000 (Invitrogen, Carlsbad, CA, USA).

### Cell viability and apoptosis assay

A CCK-8 assay kit (Beyotime Biotechnology, Shanghai, China) was used to determine cell viability by measuring absorbance at 450 nm using a microplate reader. Cell apoptosis was determined using an FITC Annexin V Apoptosis Detection Kit or a PE Annexin V Apoptosis Detection Kit (556547 and 559763, respectively; BD Biosciences). Briefly, cells were cultured in a six-well plate overnight, and the treated cells were collected and labelled with annexin V and propidium iodide or annexin V and 7-Amino-Actinomycin D for 15 min in the dark. Apoptotic cells were analysed using flow cytometry.

### Biochemical analysis

We analysed the serum and urine samples using automatic biochemistry. Serum creatinine (Scr) and blood urea nitrogen (BUN) levels were measured using assay kits (Jiancheng Biotech, Nanjing, China).

### Histological examination

Kidney tissues were dissected and fixed in 4% paraformaldehyde, embedded in paraffin, sliced into 3 µm sections, and stained with haematoxylin-eosin (HE). For immunohistochemical staining, kidney sections were deparaffinised and rehydrated, and antigens were retrieved with citrate buffer. After blocking with sheep serum for 30 min, the sections were incubated with diluted primary antibodies in skimmed milk (Kim-1 and 4HNE) overnight at 4 °C. A horseradish peroxide (HRP)-conjugated goat anti-rabbit was used as the secondary antibody. Images of stained sections were viewed under a microscope (Eclipse 80i; Nikon, Tokyo, Japan) with a digital camera (DS-U1; Nikon). The primary antibodies used for immunohistochemistry staining are shown in Supplementary Table S2. Apoptosis of renal sections was evaluated by TUNEL staining kit (Beyotime Biotechnology) according to the manufacturer’s protocol.

### Transmission electron microscopy

The kidney tissues were fixed in 2.5% glutaraldehyde at pH 7.43, dehydrated, and embedded in Epon. The embedded tissues were cut into ultrathin sections, stained with 5% uranyl acetate and lead citrate, and analysed using transmission electron microscopy (TEM) (JEM-1400plus; JEOL, Tokyo, Japan).

### Western blot analysis

Renal cortex and HK-2 cells were lysed in RIPA buffer with a protease inhibitor and quantified using the bicinchoninic acid (BCA) method (Solarbio). After electrophoresis on 10% SDS–PAGE gels, the samples were transferred to polyvinylidene difluoride membranes (0.45 μm), blocked with 5% skimmed milk, and incubated with the primary antibodies at 4 °C overnight and secondary antibodies at room temperature for 1 h. Immunoblotting signals were detected using an enhanced chemiluminescence detection system (Tanon 5200; Shanghai, China). β-actin was used as an internal reference protein. Quantification was performed using ImageJ software (NIH, USA). The primary antibodies used are shown in Supplementary Table S2.

### Co-immunoprecipitation

Pierce™ Protein A/G Magnetic Beads (Thermo Fisher Scientific, Waltham, MA, USA) were incubated with anti-PGC1α or anti-FLAG antibodies and rotated at room temperature for 1 h. Cell lysates were added to the cross-linked magnetic beads and incubated for 1 h at room temperature on a rotating platform. Subsequently, the magnetic bead complex was separated using a magnetic stand and washed twice with the lysis buffer. The pellet was then washed with elution buffer, boiled in sodium dodecyl sulphate-polyacrylamide gel electrophoresis loading buffer, and subjected to western blot analysis. The primary antibodies used were shown in Supplementary Table S2.

### Quantitative real-time PCR

TRIzol reagent (Invitrogen, Waltham, MA, USA) and Transcriptor Reverse Transcriptase (RR036A; Takara, Kusatsu, Japan) were used for total RNA isolation and complementary DNA (cDNA) synthesis. Real-time PCR (RT-PCR) analysis was performed in triplicate using a SYBR Green PCR kit (RR820A, Takara) on a CFX96 RT-PCR detection system (Bio-Rad, Hercules, CA, USA). The primer sequences of the target mRNA and internal control (β-actin) are listed in Supplementary Table S1.

### Mitochondrial DNA copy measurement

Total DNA was isolated from the kidneys using a DNA extraction kit (Tiangen, Beijing, China), according to the manufacturer’s protocol. The relative mitochondrial DNA (mtDNA) content was assessed by quantitative RT-PCR using primers for mitochondria-encoded NADH dehydrogenase 1 (ND-1) and normalised to nuclear-encoded β-actin. Primer sequences for ND-1 and β-actin are listed in Table S1.

### Immunofluorescence

Cells were washed in phosphate-buffered saline (PBS) three times, incubated with 200 nM MitoTracker Deep Red (Beyotime Biotechnology) at 37 °C for 30 min, fixed with 4% paraformaldehyde for 30 min, and permeabilised with 0.1% Triton X-100 for 10 min. After blocking with 1% BSA for 1 h, the cells were incubated with diluted primary antibody (ALDH2 at 1:200 ratio) overnight at 4 °C. After washing, the cells were incubated with the corresponding FITC-conjugated secondary antibodies (1:200, 1 h at 37 °C). The nuclei were stained with 4′,6-diamidino-2-phenylindole for 5 min at room temperature. Immunofluorescence micrographs were captured using Nikon AXR confocal laser microscope.

### Mitochondrial membrane potential

The mitochondrial membrane potential (ΔΨ m) was detected by JC-1 (Beyotime Biotechnology). Briefly, cells were washed twice in PBS, stained with JC-1 for 20 min at 37 °C, washed twice with binding buffer, and imaged using a Nikon AXR confocal laser microscope.

### Seahorse XFe96

The mitochondrial oxygen consumption rate (OCR) and extracellular acidification rate (ECAR) were measured using a Seahorse XFe96 Flux Analyzer (Seahorse Biosciences, Agilent, Santa Clara, CA, USA) with the Mito Stress Test kit and Glycolysis Stress Test kit. Cells (2 × 10^3^ per well) were plated in XF-96 extracellular flux assay plates in 80 μL of DMEM-F12 medium. After overnight incubation at 37 °C, the medium was replaced with XF DMEM (pH 7.4). For the Mito stress test, the test compounds were added in the following order: oligomycin (1.5 μM), FCCP (1.0 μM), and rotenone/antimycin A (both 0.5 μM). For the glycolysis stress test, test compounds were added in the following order: glucose (10 mM), oligomycin (1.5 μM), and 2-DG (50 mM). The values obtained in each measurement were averaged and normalised by the cells number of per well (Cytation 7, Cell Imaging Multi-Mode Reader, Agilent BioTek, VT).

### Measurement of lactate and ATP

Lactate in the kidney tissue was measured using a lactate detection kit (Solarbio) according to the manufacturer’s instructions. Tissues were lysed with extraction buffer, and the supernatant was used to measure lactate levels. The lactate content of the kidney was normalised by tissue weight.

ATP levels were measured using an ATP assay kit (Beyotime Biotechnology), according to the manufacturer’s instructions. First, we collected tissue lysates with lysis buffer, centrifuged at 12,000 *g* for 5 min at 4 °C, and then transferred the supernatant and luciferase reagents to a 96-well plate. Luminescence was detected using a multifunctional microplate reader (Thermo Fisher Scientific) and ATP levels were normalised by protein concentration.

### mRNA sequencing

RNA integrity was assessed using the RNA Nano 6000 Assay Kit of the Bioanalyzer 2100 system (Agilent Technologies), and the index-coded samples were clustered on a cBot Cluster Generation System using the TruSeq PE Cluster Kit v3-cBot-HS (Illumina, San Diego, CA, USA), according to the manufacturer’s instructions. The library preparations were sequenced on an Illumina NovaSeq platform to generate 150 bp paired-end reads for differential expression and enrichment analysis.

### Statistical analysis

Statistical analyses were performed using GraphPad Prism 8.0 software, and quantitative data were presented as the mean ± S.E.M. Differences between groups were analysed for statistical significance by Student’s two-tailed unpaired *t*-test, one-way or two-way ANOVA. Statistical significance was set at *P* < 0.05.

## Results

### ALDH2 activation attenuated renal injury in cisplatin-induced AKI

**Fig. 1:**
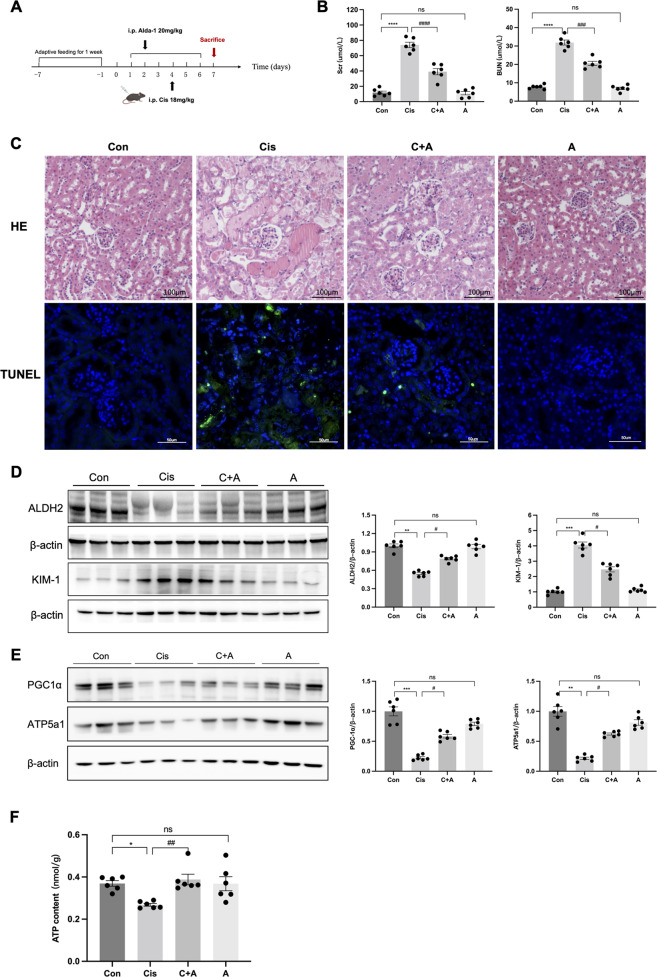
ALDH2 activation attenuated renal injury in cisplatin-induced AKI. **A** Mice were intraperitoneally (i.p.) injected with a single dose of cisplatin (18 mg/kg) and were sacrificed on the 3rd day; Alda-1 (ALDH2 agonist, 20 mg/kg) was injected by i.p. for 6 days. **B** Serum creatinine (Scr) and urea nitrogen (BUN) levels were measured in each group (*n* = 6). **C** Images of hematoxylin-eosin (HE) staining (*n* = 6). Scale bars, 100 μm; Terminal deoxynucleotidyl transferase–mediated dUTP nick end-labelling (TUNEL) assay were used to evaluate the tubular apoptosis (*n* = 6). Scale bars, 50μm. **D** The expression of ALDH2 and KIM-1 was measured by western blotting (*n* = 6). **E** The expression of mitochondria-related proteins (PGC-1α and ATP5a1) was measured by western blotting (*n* = 6). **F** ATP levels were assessed using an ATP Assay Kit (*n* = 6). *P < 0.05, **P < 0.01, ***P < 0.001, ****P < 0.0001; ^#^*P* < 0.05, ^##^*P* < 0.01, ^###^*P* < 0.001, ^####^*P* < 0.0001, ns not significant. (Con Control, Cis cisplatin, C + A Cisplatin+Alda-1, A Alda-1).

The decreased expression of ALDH2 was significant in the cisplatin-induced AKI (Cis-AKI) mice by western blot. ALDH2 agonist Alda-1 prevented the increase of Scr (38.54 ± 4.92 versus 74.42 ± 4.39 μmol/L, *P* < 0.0001) and BUN (20.56 ± 0.34 versus 31.88 ± 1.68 μmol/L, *P* < 0.001) compared to Cis-AKI mice, and alleviated the renal proximal tubular histopathological injury, with partially increased ALDH2 levels (Fig. 1A–D). Kidney injury molecule-1 (KIM-1) levels were significantly higher in the Cis-AKI group than in the Alda-1 pre-treatment group (Fig. 1D). In addition, mitochondrial-related proteins (PGC-1α and ATP5a1) were downregulated as ATP content in the renal cortex decreased in Cis-AKI mice, while they were partially restored in the Alda-1 pre-treatment group (Fig. 1E, F).

### *ALDH2*-deficient mice exhibited aggravated renal injury in cisplatin-induced AKI

**Fig. 2:**
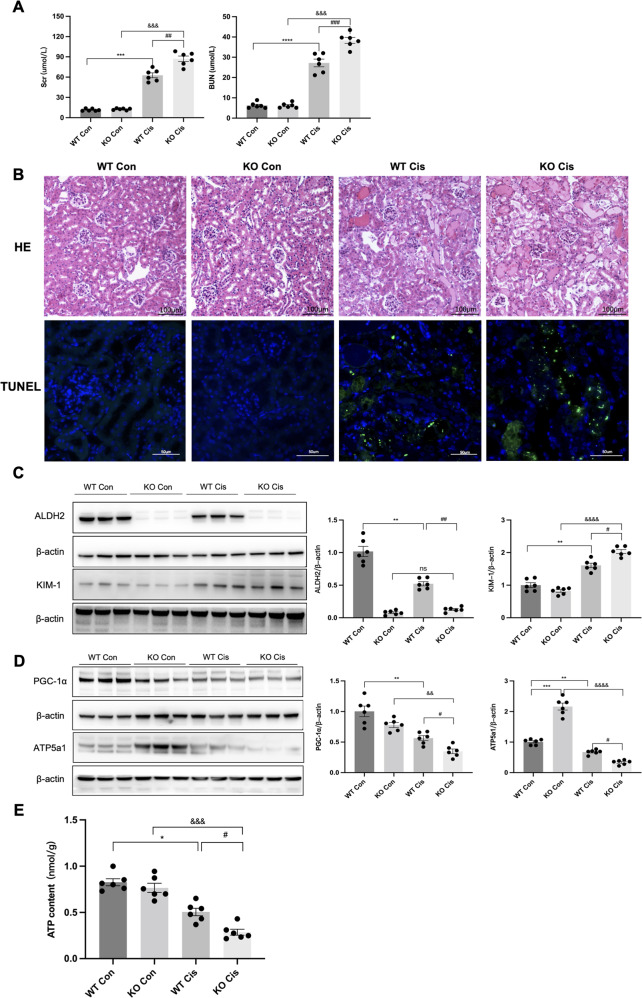
*ALDH2* knockout aggravated renal injury in cisplatin-induced AKI. **A** Serum creatinine (Scr) and urea nitrogen (BUN) levels were measured in each group (*n* = 6). **B** Images of hematoxylin-eosin (HE) staining (*n* = 6). Scale bars, 100 μm; Terminal deoxynucleotidyl transferase–mediated dUTP nick end-labelling (TUNEL) assay were used to evaluate the tubular apoptosis (*n* = 6). Scale bars, 50 μm. **C** The expression of ALDH2 and KIM-1 was measured by western blotting (*n* = 6). **D** The expression of mitochondria-related proteins (PGC-1α and ATP5a1) was measured by western blotting (*n* = 6). **E** ATP levels were assessed using an ATP Assay Kit (n = 6). *P < 0.05, **P < 0.01, ***P < 0.001, ****P < 0.0001; ^#^*P* < 0.05, ^##^*P* < 0.01, ^###^*P* < 0.001, ^####^*P* < 0.0001; ^&^*P* < 0.05, ^&&^*P* < 0.01, ^&&&^*P* < 0.001, ^&&&&^*P* < 0.0001; ns not significant. (WT Con wild-type control, KO Con knockout control, WT Cis wild-type cisplatin, KO Cis knockout cisplatin).

To assess the role of ALDH2 in Cis-AKI, *ALDH2* KO mice were intraperitoneally injected with cisplatin. In *ALDH2* KO mice with Cis-AKI, the Scr (86.13 ± 6.20 versus 62.29 ± 3.19 μmol/L, *P* < 0.01), BUN (39.78 ± 1.42 versus 27.13 ± 2.25 μmol/L, *P* < 0.001), and tubular injury were more severe than wild-type (WT) Cis-AKI mice (Fig. 2A, B). Consistent with the results of renal function, KIM-1 was increased in Cis-AKI mice and to a higher level in *ALDH2* KO Cis-AKI mice (Fig. 2C). In addition, the expression of mitochondrial-related proteins (PGC-1α and ATP5a1) and ATP levels decreased significantly in *ALDH2* KO Cis-AKI mice compared to those in WT Cis-AKI mice (Fig. 2D, E).

### Activation of ALDH2 alleviates MA-induced renal injury in mice

**Fig. 3:**
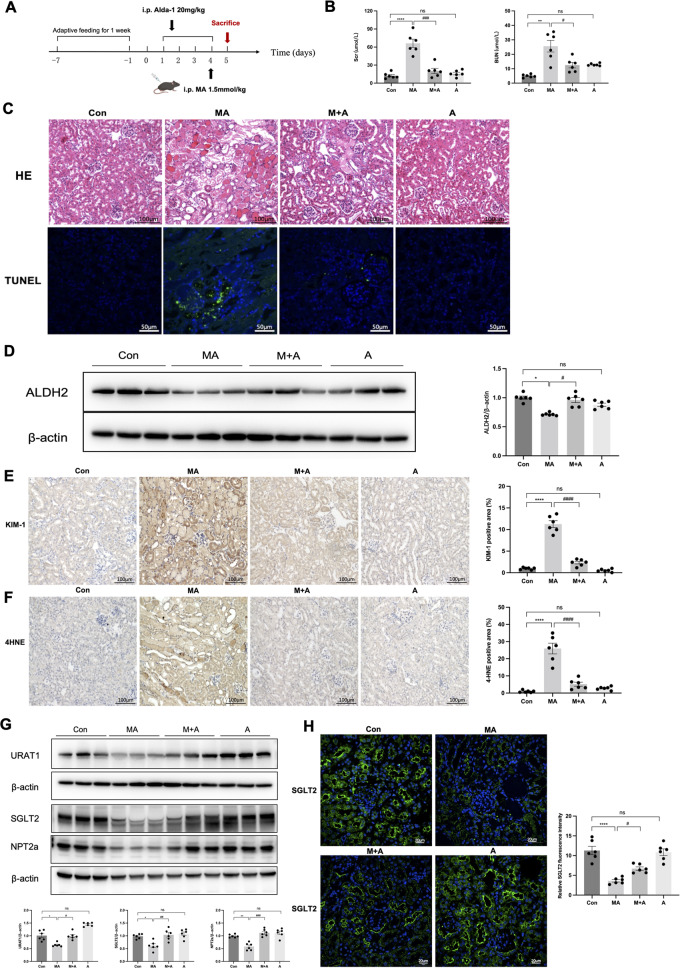
ALDH2 activation alleviated renal injury in MA-induced AKI. **A** Mice were intraperitoneally (i.p.) injected with a single dose of maleic acid (MA) (1.5 mmol/kg) and were sacrificed after 24 h; Alda-1 (ALDH2 agonist, 20 mg/kg) was injected by i.p. for 4 days. **B** Serum creatinine (Scr) and urea nitrogen (BUN) levels were measured in 4 groups (*n* = 6). **C** Images of hematoxylin-eosin (HE) staining (*n* = 6). Scale bars, 100 μm; Terminal deoxynucleotidyl transferase–mediated dUTP nick end-labelling (TUNEL) assay were used to evaluate the tubular apoptosis (*n* = 6). Scale bars, 50 μm. **D** Western blotting and the quantitative analysis of ALDH2 (*n* = 6). **E**, **F** Representative images of immunohistochemical staining of KIM-1 and 4HNE and quantitative analysis (*n* = 6). **G**, **H** The expression of tubular transporter proteins (SGLT2, NPT2a and URAT1) was measured by western blotting (*n* = 6) and immunofluorescence (*n* = 6). Scale bars, 20 μm. *P < 0.05, **P < 0.01, ***P < 0.001, ****P < 0.0001; ^#^*P* < 0.05, ^##^*P* < 0.01, ^###^*P* < 0.001, ^####^*P* < 0.0001; ns not significant. (Con Control, MA maleic acid, M + A maleic acid + Alda-1, A Alda-1).

MA is well known to cause mitochondrial dysfunction in the PTCs. To explore the effects of ALDH2 on mitochondrial dysfunction, we established a MA-induced AKI with FS mice model. Renal injury was evident on the day one and recovered on day seven (Figure S2). The decreased expression of ALDH2 was accompanied by increased Scr and BUN, renal histopathological injury, and PTC apoptosis in the MA-induced mouse model (Fig. 3A–D). The renal function, indicated by Scr (20.09 ± 3.65 versus 64.55 ± 12.64 μmol/L, *P* < 0.001) and BUN (12.52 ± 1.92 versus 25.63 ± 5.05 μmol/L, *P* < 0.05), was recovered by Alda-1 pre-treatment with reduced KIM-1 and 4-hydroxynonenal (4-HNE) compared with those in MA-induced mice (Fig. 3E, F). Furthermore, we evaluated the changes of tubular transporter proteins, which are damaged in FS. Western blotting showed that the expression of tubular transporter proteins significantly declined in MA-induced mice, but recovered partially after Alda-1 pre-treatment (Fig. 3G, H).

### ALDH2 improved mitochondrial homoeostasis in MA-induced mice

**Fig. 4:**
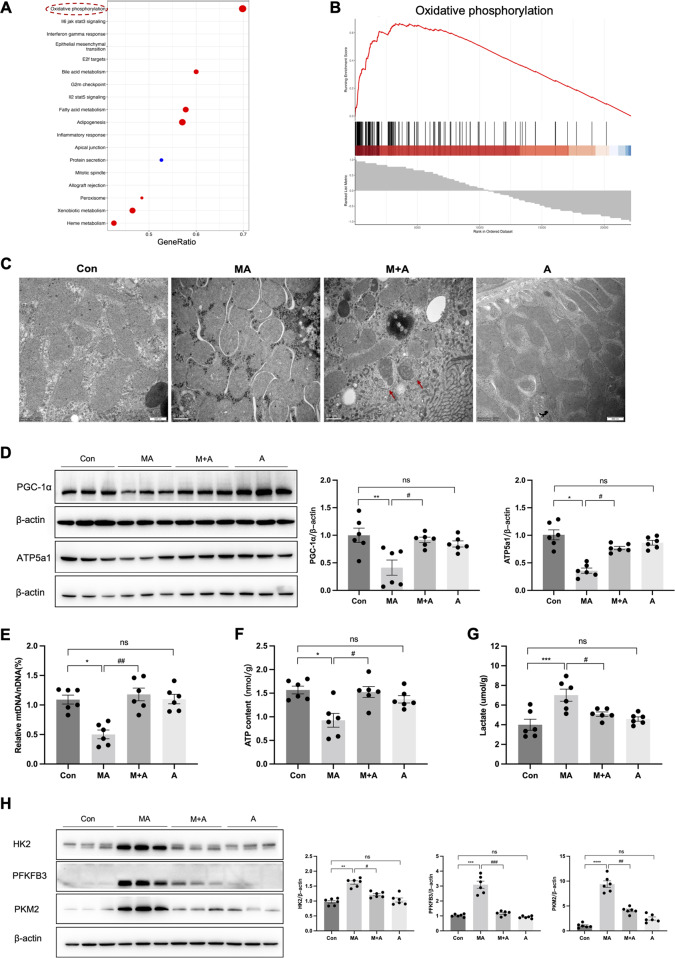
ALDH2 activation restored mitochondrial homoeostasis and suppressed aerobic glycolysis in MA-induced AKI. **A** Gene set enrichment analysis (GSEA) analysis for differentially expressed genes (DEGs) between MA and M + A groups. **B** GSEA analysis of the oxidative phosphorylation pathway. **C** Representative transmission electron microscopy (TEM) micrographs of mouse renal tubular epithelial cell mitochondria in each group. The red arrow showed autophagosomes. Scale bars, 0.5μm. **D** The expression of mitochondria-related proteins (PGC-1α and ATP5a1) was measured by western blotting (*n* = 6). **E** mtDNA content was assessed by quantitative RT-PCR expressed as the ratio of the mitochondrial genome to the nuclear genome (*n* = 6). **F** ATP levels were assessed using an ATP Assay Kit (*n* = 6). **G** The lactate (the final product of aerobic glycolysis) content was measured in 4 groups (*n* = 6). **H** The expression of aerobic glycolysis-related enzymes (HK2, PFKFB3 and PKM2) was measured by western blotting (*n* = 6). *P < 0.05, **P < 0.01, ***P < 0.001, ****P < 0.0001; ^#^*P* < 0.05, ^##^*P* < 0.01, ^###^*P* < 0.001, ^####^*P* < 0.0001; ns not significant. (Con Control, MA maleic acid, M + A maleic acid + Alda-1, A Alda-1, mtDNA mitochondrial DNA, nDNA nuclear DNA).

To further investigate the mechanism by which ALDH2 alleviates MA-induced renal damage, we performed a transcriptomic analysis of the renal cortex of MA-induced mice with or without Alda-1 pre-treatment. Gene set enrichment analysis (GSEA) revealed that the oxidative phosphorylation pathway was positively enriched after Alda-1 pre-treatment (Fig. 4A, B). Consistently, TEM showed swollen mitochondria and ruptured mitochondrial cristae in MA-induced AKI (MA-AKI) mice, which were obviously attenuated by ALDH2 activation with many autophagosomes observed in the tubular cells (Fig. 4C). More importantly, MA-AKI mice showed decreased levels of mitochondrial-related proteins (PGC-1α and ATP5a1), mtDNA, and ATP content, but this effect was reversed by pre-treatment with Alda-1 (Fig. 4D–F).

To assess mitochondrial energy metabolism after ALDH2 activation, we evaluated the glycolytic capacity, which is a complementary energy supply after mitochondrial dysfunction. In MA-AKI mice, levels of glycolysis-related enzymes (HK2, PFKFB3, and PKM2) and the final product (lactate) were increased, whereas they were suppressed by Alda-1 pre-treatment (Fig. 4G, H).

### ALDH2 improved mitochondrial dysfunction and apoptosis in MA-treated HK-2 cells

**Fig. 5:**
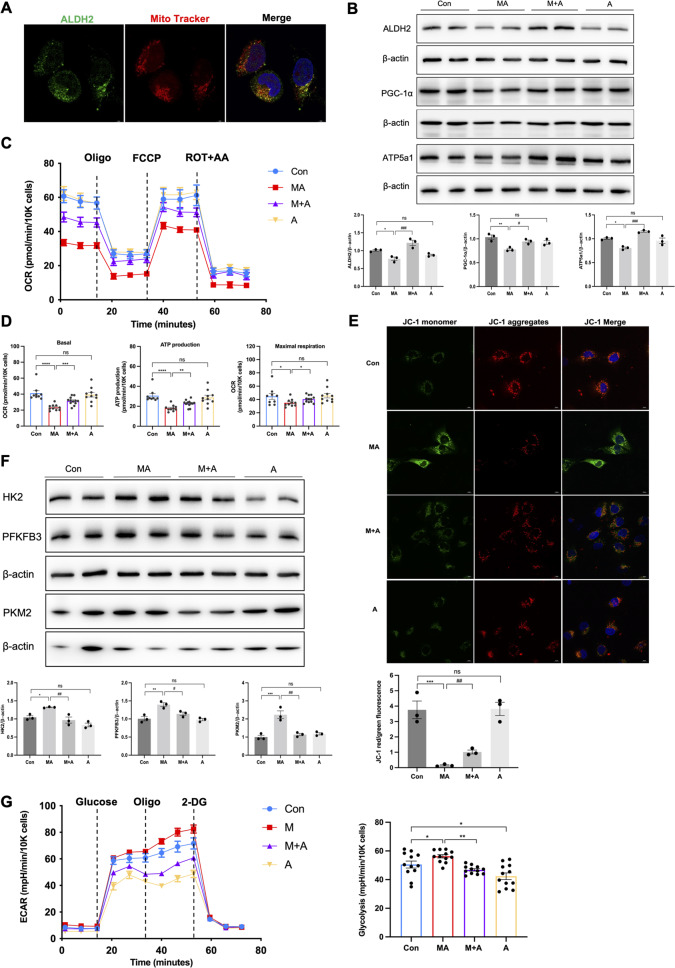
ALDH2 activation attenuated mitochondrial dysfunction in MA-treated HK-2 cells. **A** Immunofluorescent analysis of the colocalization between ALDH2 and mitochondria (indicated by Mito Tracker) in HK-2 cells (*n* = 3). Scale bars, 20 μm. **B** The expression of mitochondria-related proteins (ALDH2, PGC-1α and ATP5a1) was measured by western blotting (*n* = 3). **C**, **D** Measurement of mitochondrial oxygen consumption ratio (OCR) in HK-2 cells (*n* = 9–12 each group). **E** Images of JC-1 staining for mitochondrial membrane potential in 4 groups (*n* = 3). Scale bars, 20 μm. **F** The expression of aerobic glycolysis-related enzymes (HK2, PFKFB3 and PKM2) was measured by western blotting (*n* = 3). **G** Measurement of mitochondrial extracellular acidification rate (ECAR) in HK-2 cells (*n* = 12 each group). *P < 0.05, **P < 0.01,***P < 0.001, ****P < 0.0001; ^#^*P* < 0.05, ^##^*P* < 0.01, ^###^*P* < 0.001, ^####^*P* < 0.0001; ns not significant. (Con Control, MA maleic acid, M + A maleic acid + Alda-1, A Alda-1, OCR oxygen consumption ratio, ECAR extracellular acidification rate, Oligo oligomycin, ROT + AA rotenone/antimycin A).

Subsequently, we evaluated the effect of ALDH2 on mitochondrial function in HK-2 cells. MitoTracker labelling showed that ALDH2 mainly colocalised with the mitochondria in HK-2 cells (Fig. 5A). The expression of ALDH2 was significantly decreased in MA-treated HK-2 cells, with inhibition of mitochondria-related proteins (PGC-1α and ATP5a1), but increased after incubation with Alda-1 (Fig. 5B). MA-treated HK-2 cells demonstrated lower baseline OCR and a reduction in FCCP-induced elevation in OCR, indicating low mitochondrial respiration activity. Activation of ALDH2 effectively improved the oxidative phosphorylation capacity, ATP production, and mitochondrial membrane potential (Fig. 5C–E).

**Fig. 6:**
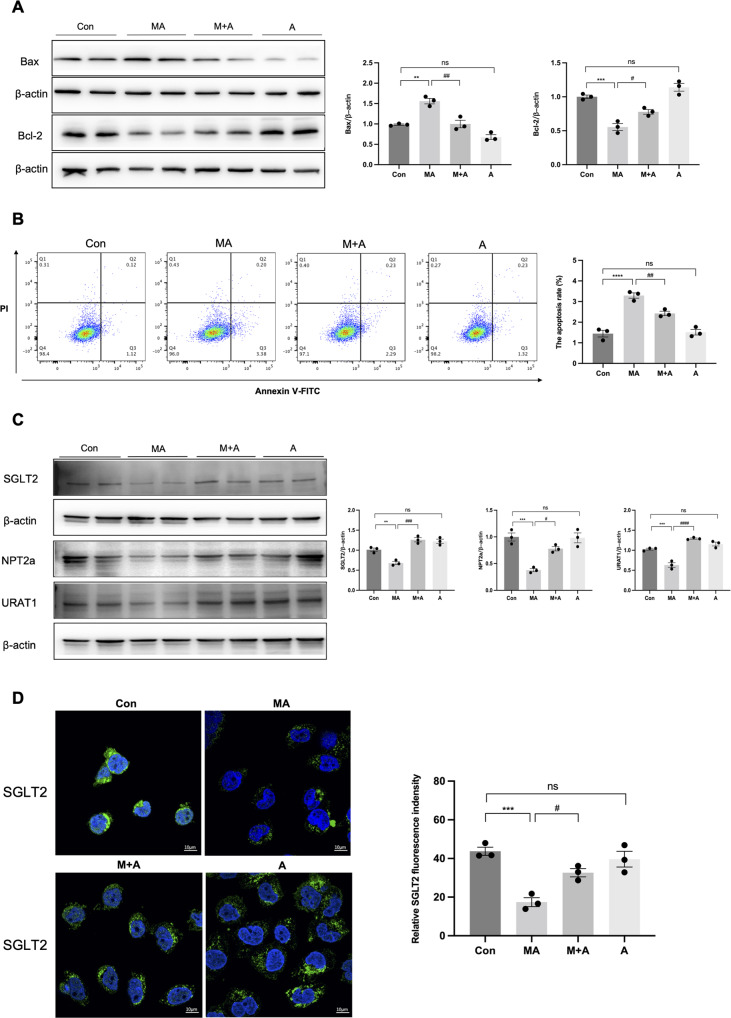
ALDH2 activation inhibited apoptosis and tubular transporter disorder in HK-2 cells. **A** The expression of apoptosis-related proteins (Bax and Bcl-2) was measured by western blotting (*n* = 3). **B** Apoptosis was determined by flow cytometry in 4 groups (*n* = 3). **C**, **D** The expression of tubular transporter proteins (SGLT2, NPT2a and URAT1) was measured by western blotting (*n* = 3) and immunofluorescence (*n* = 3). Scale bars, 10 μm. *P < 0.05, **P < 0.01, ***P < 0.001, ****P < 0.0001; ^#^*P* < 0.05, ^##^*P* < 0.01, ^###^*P* < 0.001, ^####^*P* < 0.0001; ns not significant. (Con Control, MA maleic acid, M + A maleic acid + Alda-1, A Alda-1).

In parallel with the in vivo findings, glycolysis was upregulated after incubation with MA but was reduced by Alda-1 treatment, as indicated by the related enzymes (HK2, PFKFB3, and PKM2; Fig. 5F). ECAR, a direct indication of glycolysis, was markedly increased in HK-2 cells after 24 h of MA treatment, whereas Alda-1 prevent the glycolysis significantly, both in MA + A and A groups (Fig. 5G). In addition, we observed increased apoptosis (3.29 ± 0.22% versus 1.44 ± 0.28%, *P* < 0.001) by flow cytometry and apoptosis markers in MA-treated HK-2 cells, which were significantly reversed (2.43 ± 0.18% versus 3.29 ± 0.22%, *P* < 0.01) after incubation with Alda-1 (Fig. 6A, B). In addition, the tubular transporter proteins decreased after MA treatment, whereas Alda-1 elevated the expression levels (Fig. 6C, D).

Conversely, sh*ALDH2*-mediated *ALDH2* knockdown aggravated mitochondrial disruption and cell apoptosis, whereas it upregulated glycolysis in MA-induced HK-2 cells (Figure S4).

### ALDH2 and PGC-1α interaction facilitated nuclear translocation of PGC-1α

**Fig. 7:**
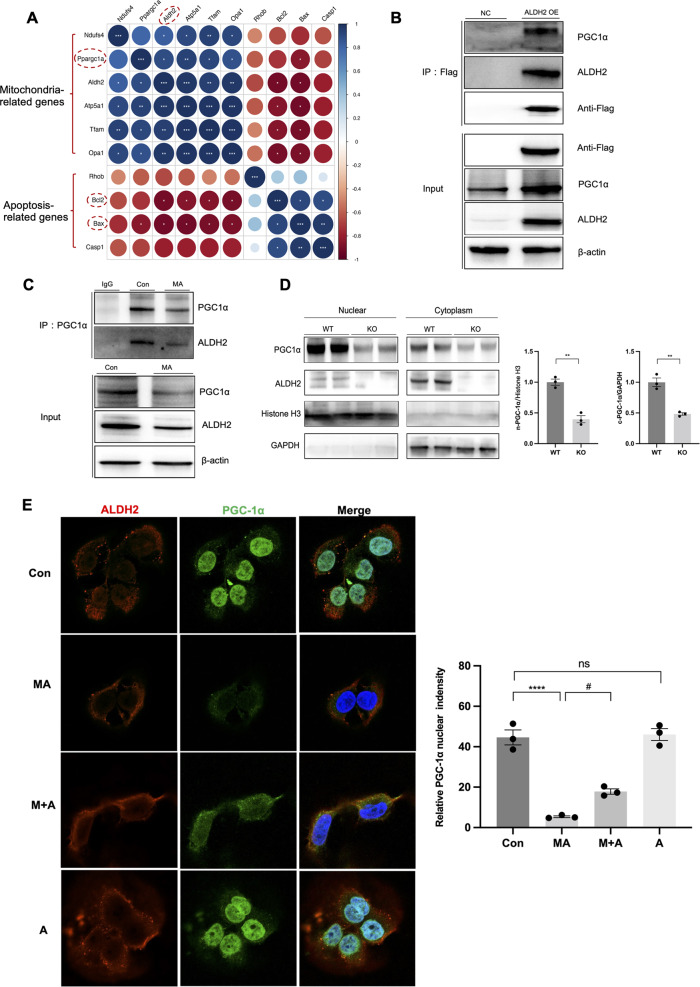
ALDH2 interacted with PGC-1α and modulated nuclear translocation of PGC-1α. **A** The heap map showing the correlations of ALDH2 and the mitochondria and apoptosis-related genes. **B** Co-immunoprecipitation assay for the interaction of ALDH2 with PGC-1α in ALDH2 overexpression HK-2 cells (*n* = 3). Input immunoblotted is shown as a control. **C** Co-immunoprecipitation analysis investigating the interaction of endogenous ALDH2 with PGC-1α in HK-2 cells with or without MA treatment (*n* = 3). Input immunoblotted is shown as a control. **D** ALDH2 enhanced nuclear translocation of PGC-1α in WT kidney tissues (WT, *n* = 3; KO, *n* = 3). **E** Immunofluorescent analysis of the location of PGC-1α and ALDH2 in HK-2 cells with or without MA and Alda-1 treatment (*n* = 3). *P < 0.05, **P < 0.01,***P < 0.001, ****P < 0.0001; ^#^*P* < 0.05. (Con Control, MA maleic acid, M + A maleic acid + Alda-1, A Alda-1, n-PGC-1α nuclear PGC-1α, c-PGC-1α cytoplasmic PGC-1α, WT wild type, KO knockout, NC normal control OE, overexpression).

Next, we investigated the mechanism by which ALDH2 modulated mitochondrial function. Heatmap analysis of the transcriptomics revealed correlations between ALDH2 and mitochondria-related genes, among which PGC-1α was significantly correlated (Fig. 7A). Co-immunoprecipitation assays demonstrated the interaction between ALDH2 and PGC-1α in *ALDH2* overexpression HK-2 cells (Fig. 7B). Endogenous association was further validated in control and MA-treated HK-2 cells. Immunoprecipitation analysis revealed that PGC-1α interacted with ALDH2, which weakened after incubation with MA (Fig. 7C). ALDH2 mainly exists in the mitochondria and cytoplasm (Fig. 5A), whereas PGC-1α is primarily located in the nucleus. To further explore the cellular locations of these protein interactions, we separated the nuclear and cytoplasmic fractions from the kidneys of the WT and *ALDH2* KO mice. ALDH2 deficiency in KO mice decreased PGC-1α in both the nucleus and cytoplasm (Fig. 7D). Consistently, immunofluorescence staining showed that nuclear and cytosolic PGC-1α decreased in MA-treated HK-2 cells, whereas ALDH2 activation significantly increased the expression of PGC-1α in the nucleus and cytoplasm (Fig. 7E).

### *PGC-1α* knockdown abolished the protective effects of ALHD2 activation in MA-treated HK-2 cells

**Fig. 8:**
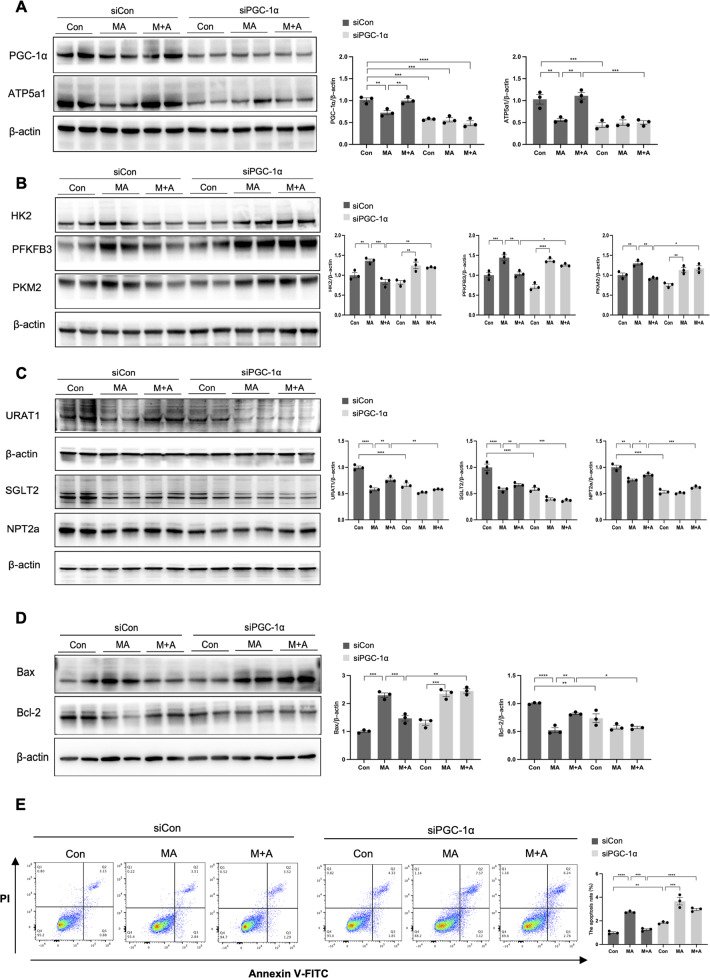
Knockdown of *PGC-1α* abrogates the improvement of ALDH2 activation on mitochondria, tubular transporter disorder and cell apoptosis in HK-2 cells. HK2 cells were transfected with control siRNA or PGC-1α siRNA for 6 h and treated with MA in the presence or absence of Alda-1 for 24 h. **A** The expression of mitochondria-related proteins (PGC-1α and ATP5a1) was measured by western blotting (*n* = 3). **B** The expression of aerobic glycolysis-related enzymes (HK2, PFKFB3 and PKM2) was measured by western blotting (*n* = 3). **C** The expression of tubular transporter proteins (SGLT2, NPT2a and URAT1) was measured by western blotting (*n* = 3). **D** The expression of apoptosis-related proteins (Bax and Bcl-2) was measured by western blotting (*n* = 3). **E** Apoptosis was determined by flow cytometry in 6 groups (*n* = 3). *P < 0.05, **P < 0.01, ***P < 0.001, ****P < 0.0001, ns not significant. (Con Control, MA maleic acid, M + A maleic acid + Alda-1, siCon control siRNA, siPGC-1α PGC-1α siRNA, PI propidium iodide).

Finally, to verify the involvement of PGC-1α in the regulation of ALDH2 on mitochondrial function, HK2 cells were transfected with siPGC-1α prior to MA and Alda-1 treatment. PGC-1α knockdown almost eliminated the beneficial effects of Alda-1 against MA, as indicated by the decreased mitochondria-related proteins, tubular transporter proteins and higher levels of glycolysis-related enzymes (Fig. 8A–C). The reduced cell apoptosis after Alda-1 treatment was abrogated following transfection with siPGC-1α (Fig. 8D, E), shown as 2.96 ± 0.16% versus 1.25 ± 0.13% (*P* < 0.0001) by flow cytometry.

## Discussion

ALDH2 is an enzyme mainly located in mitochondria, involved in alcohol metabolism and oxidative stress [25]. Around 30–50% of Asians carry the rs671 mutation of *ALDH2*, leading to reduced enzyme activity to metabolise acetaldehyde [26]. Numerous studies have confirmed that *ALDH2* mutation plays a critical role in alcohol use disorders, cancer, cardiovascular diseases, diabetes mellitus, and neurodegenerative diseases [20, 27, 28]. In addition, ALDH2 attenuates reactive aldehydes and mitochondrial ROS both in vivo and in vitro, protecting against myocardial, pulmonary, hepatic events, and stroke [20, 29–31]. However, the effects of ALDH2 on mitochondrial homoeostasis in AKI have not been clarified. This study established the crucial role of ALDH2 on mitochondrial function in the progression of AKI. We demonstrated that the activation of ALDH2 attenuated cisplatin- and MA-induced tubular injury while improving mitochondrial structure, mitochondrial membrane potential, and respiration rate, which were aggravated in *ALDH2*-deficient mice. Mechanistically, ALDH2 facilitated mitochondrial biogenesis by interacting with PGC-1α and promoting its nuclear translocation.

Previous studies have confirmed that ALDH2 activation exerts a protective role in AKI by inhibiting oxidative stress, inflammatory infiltration, and autophagy [21–23]. Consistently, our study confirmed that ALDH2 exerts its protective effect by activating autophagy, as indicated by the increased autophagosomes detected by TEM (Fig. 4C). Western blot analysis also indicated that LC3B level was significantly higher in renal cortex after Alda-1 pre-treatment, while p62 level was lower. Furthermore, the autophagic inhibitor Baf A1 was used to confirm the changes of autophagic flux (Figure S5).

Renal PTCs are rich in mitochondria to provide enough energy to maintain their normal active transport function. Defects in mitochondrial dynamics and excessive mitochondrial oxidative stress contribute to AKI, which can be treated with mitochondria-targeted therapeutic strategies [32, 33]. In this study, we found that ALDH2 activation substantially improved mitochondrial morphology, mtDNA depletion, and ATP production in MA-induced AKI with suppressed glycolysis. Conversely, ALDH2 deficiency aggravated renal injury and cell apoptosis by disrupting mitochondrial homoeostasis. The specific protective effect of ALDH2 on mitochondrial function was confirmed in HK-2 cells by activation and knockdown of ALDH2. Although ALDH2 protects against oxidative stress-induced organ damage by detoxifying endogenous aldehydes such as 4-HNE and malondialdehyde (MDA) produced by lipid peroxidation [34], this is the first study to show that ALDH2 maintains mitochondrial homoeostasis and energy metabolism, highlighting a novel regulatory mechanism in AKI.

The most intriguing finding of our study is that ALDH2 promotes mitochondrial biogenesis by interacting with PGC-1α and advances its nuclear translocation, thereby mitigating AKI progression. PGC-1α is highly expressed in proximal tubules, where mitochondria are abundant. As a master regulator of mitochondrial biogenesis, PGC-1α directly regulates an array of transcription factors to modulate nuclear genes related to this process [35]. Numerous studies have shown that the loss of PGC-1α contributes to AKI and subsequent chronic kidney disease. In animal models of kidney IRI or cisplatin-induced AKI, PGC-1α decreased proportionally to the degree of kidney injury, while pharmacological activation or transgenic expression of PGC-1α improved mitochondrial function and renal injury [36, 37]. Our bioinformatic analysis suggested that ALDH2 is positively correlated with PGC-1α at the transcriptional level and this interaction was confirmed by co-immunoprecipitation. We found that ALDH2 activation increased PGC-1α expression, both at transcriptional and protein half-life regulatory levels (Figure S6). In contrast, the knockdown of *PGC-1α* abolished the protective effects of ALDH2 activation on mitochondrial homoeostasis, tubular transporter disorder, and cell apoptosis. However, the mechanism underlying the regulation of PGC-1α-mediated mitochondrial biogenesis remains unclear.

PGC-1α is predominantly distributed in the nucleus and activates transcription factors that transactivate the nuclear genes for mitochondrial biogenesis. Interestingly, PGC-1α was previously reported to change its subcellular distribution from the nucleus to the cytoplasm after exposure to toxins, which is potentially linked to disabling deacetylation [38]. Here, we found that ALDH2 deficiency decreased PGC-1α in both the nucleus and cytoplasm, whereas ALDH2 activation promoted the nuclear translocation of PGC-1α. Moreover, another study has shown that ALDH2 interacted with a poly (ADP-ribose) polymerase and attenuated its nuclear translocation, leading to higher HDL cholesterol level [39]. Although the mechanism involved in ALDH2-mediated PGC-1α nuclear translocation requires further investigation, our observations uncover a novel regulatory mechanism for ALDH2 interaction with PGC-1α.

In summary, our research revealed that ALDH2 activation alleviated mitochondrial dysfunction in AKI by enhancing PGC-1α-mediated mitochondrial biogenesis. These findings could shed new light on therapeutic strategies for AKI.

## Supplementary information

Supplementary file

Full length WB

Checklist

## Data Availability

The data supporting the findings of the present study are available from the corresponding author upon reasonable request.
