# Supplementary material for: Aldehyde dehydrogenase 2 alleviates mitochondrial dysfunction by promoting PGC-1α-mediated biogenesis in acute kidney injury
Source: Cell Death Dis. 2023 Jan 20;14(1):45. doi: 10.1038/s41419-023-05557-x (PMC9860042; doi:10.1038/s41419-023-05557-x)
Supplement: Supplementary file 1 — Supplementary file [file 41419_2023_5557_MOESM1_ESM.pdf]

Figure S1

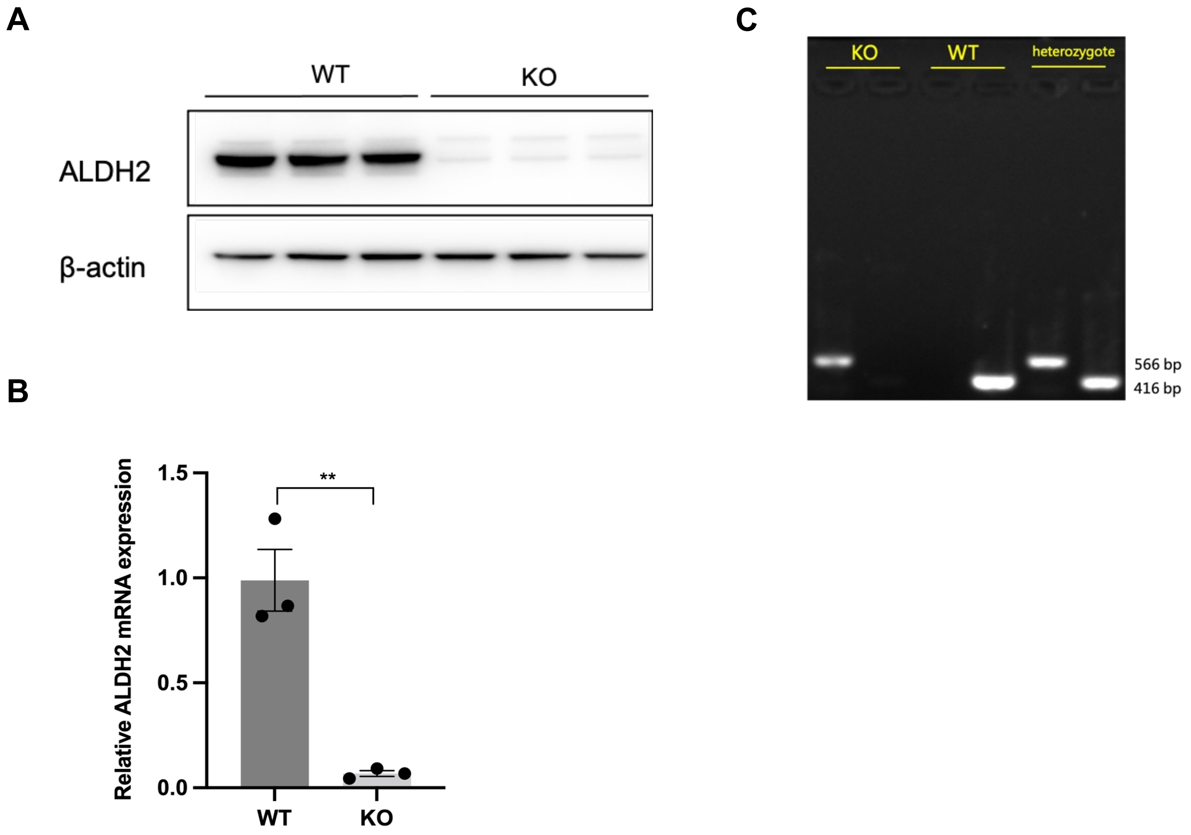

Figure S1 The genotyping for ALDH2 KO mice. (A) Western blot analysis of ALDH2 in WT and KO mice (WT, n = 3; KO, n = 3). (B) Relative mRNA expression of ALDH2 in WT and KO mice (n=6). (C) Images of PCR analysis genotyping the WT, ALDH2 KO and heterozygote mice. (KO, knockout; WT, wild type)

The following primers were used for genotyping.

Primers 1 (product size: 566 bp):

F1: 5’-GACCTATAGAATTCAGCAAACGGA-3’

R1: 5’-GAAGACTGTCAACTGTGAAAGACAG-3’

Primers 2 (product size: 416 bp):

F1: 5’-GACCTATAGAATTCAGCAAACGGA-3’

R2: 5’-CAGGTTAGAAGCACAAGTCTAGC-3’

KO is one band with 566 bp, WT is one band with 416 bp and heterozygous is two bands with 566 bp and 416 bp.

Figure S2

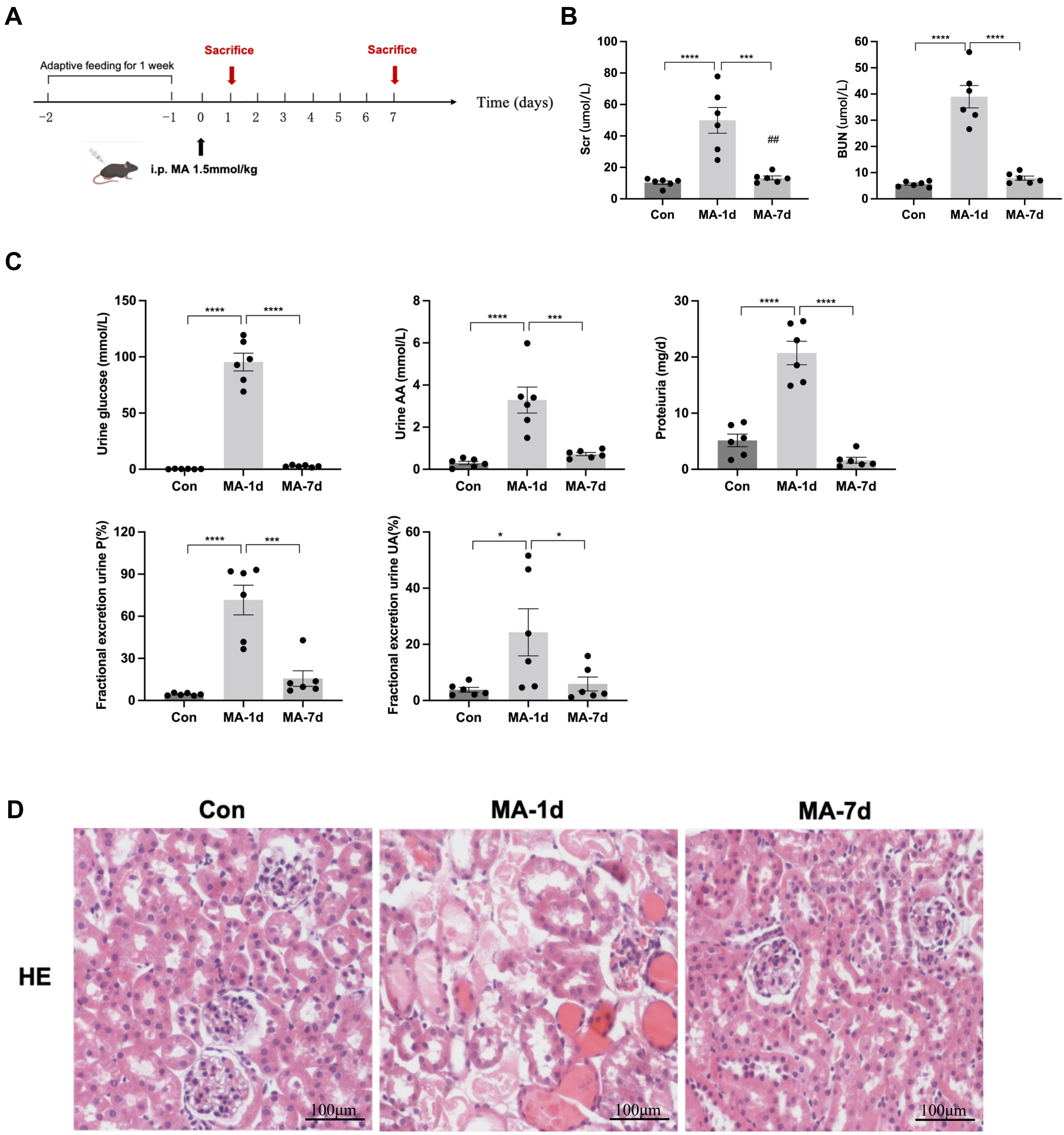

Figure S2 Maleic acid (MA) induced an experimental model of Fanconi syndrome in mice. (A) Mice were intraperitoneally injected with a single dose of MA (1.5mmol/kg) and were sacrificed on the 1st day and 7th day. (B) Scr and BUN levels were measured in each group (n=6). (C) The criterion of Fanconi syndrome was evaluated in each group (n=6). (D) Images of HE staining (n=6). Scale bars, 100μm. **P* < 0.05, ** *P* < 0.01, *** *P* < 0.001, **** *P* < 0.0001. (Con, Control; MA, maleic acid).

Figure S3

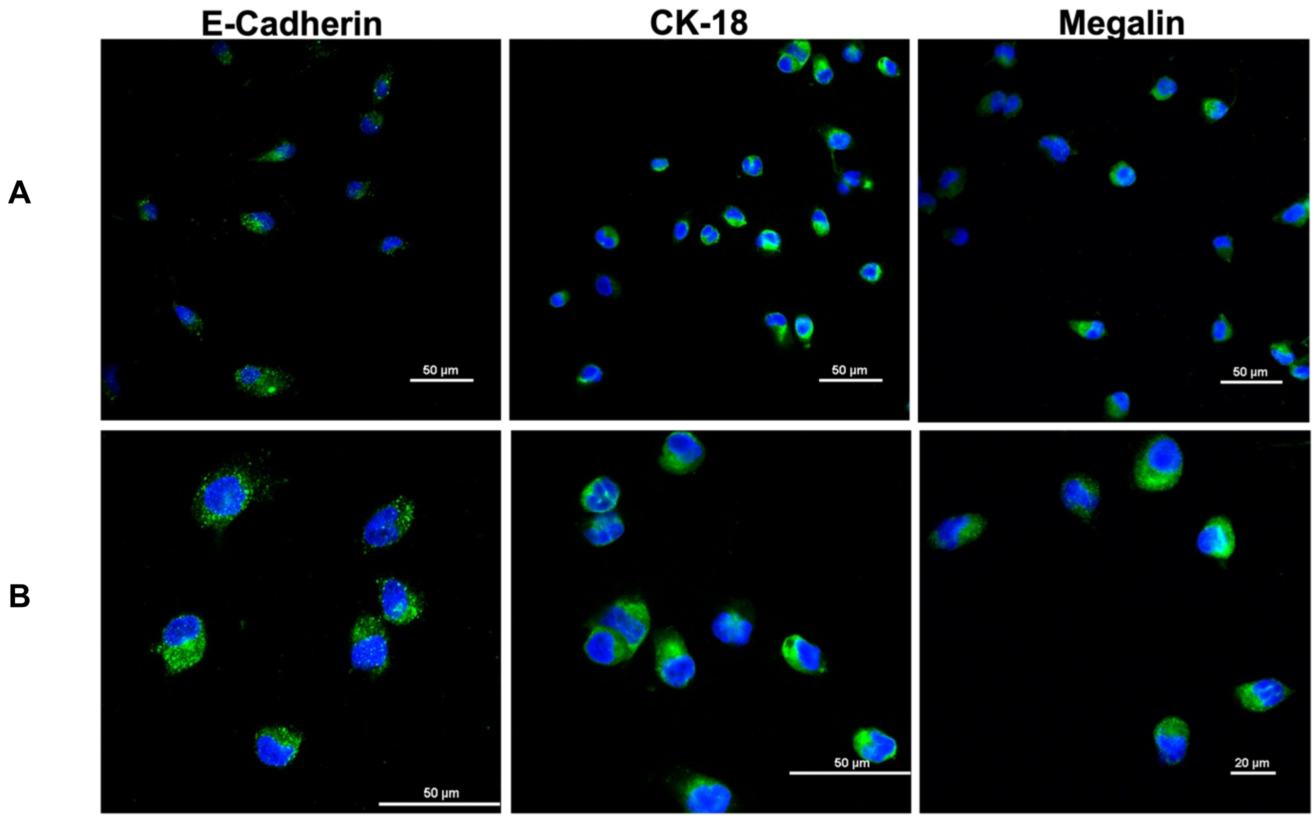

Figure S3 Identification of HK-2 cells. (A-B) Immunofluorescence of HK-2 cells markers. Scale bars, 50µm.

Figure S4

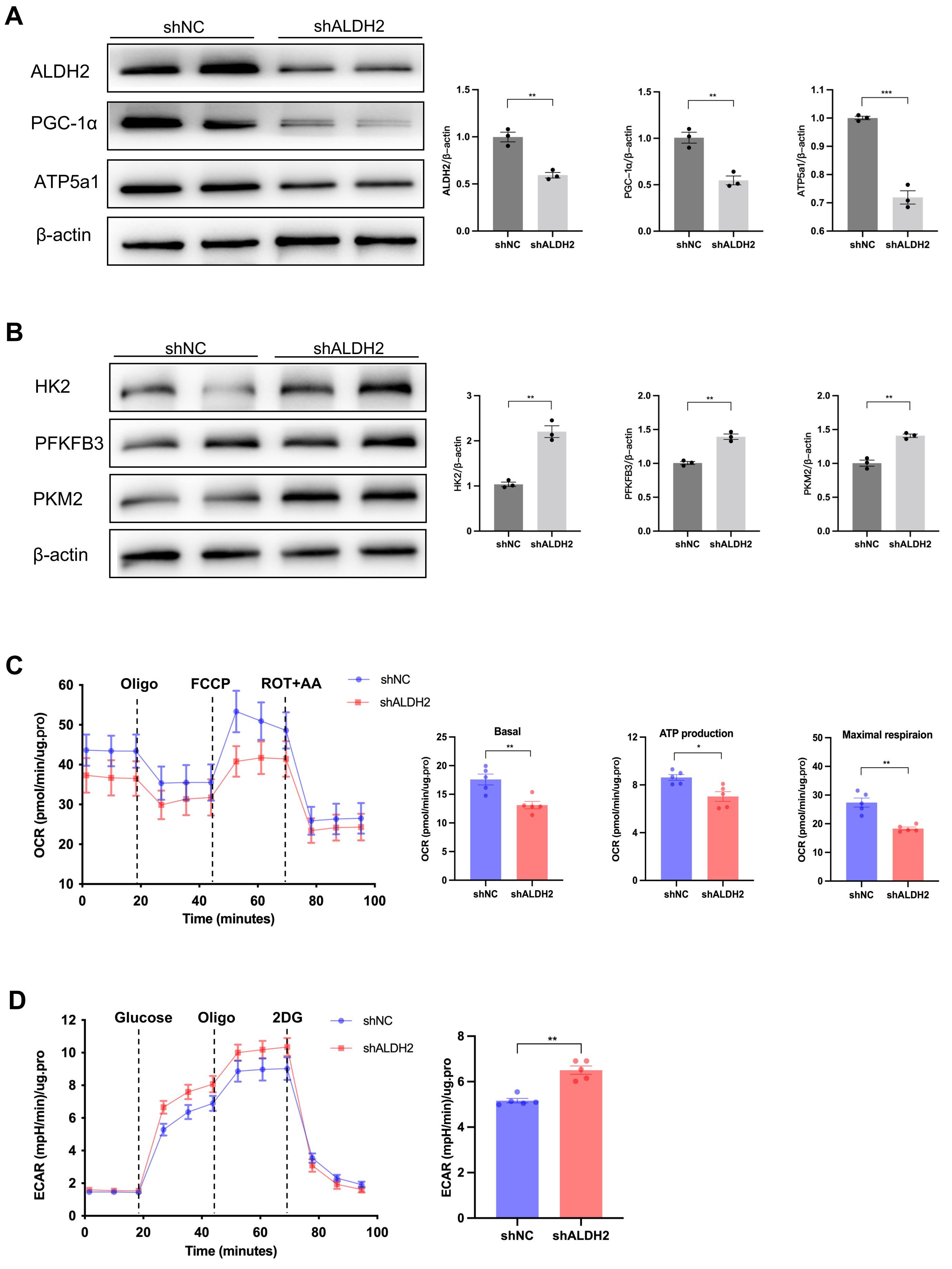
A
shNC
shALDH2
ALDH2
PGC-1α
ATP5a1
β-actin
ALDH2/β-actin
PGC-1α/β-actin
ATP5a1/β-actin
B
HK2
PFKFB3
PKM2
HK2/β-actin
PFKFB3/β-actin
PKM2/β-actin
C
Oligo
FCCP
ROT+AA
OCR (pmol/min/ug.pro)
Time (minutes)
Basal
ATP production
Maximal respiraion
D
Glucose
Oligo
2DG
ECAR (mpH/min)/ug.pro

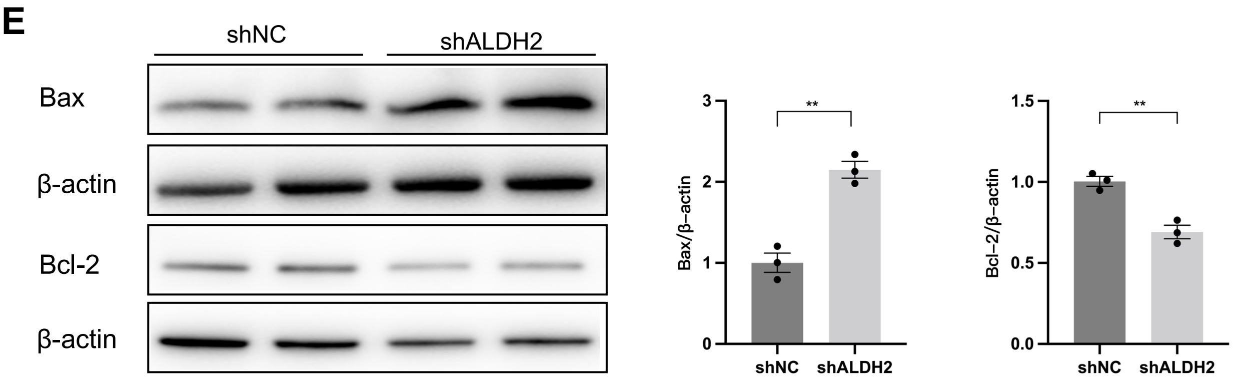

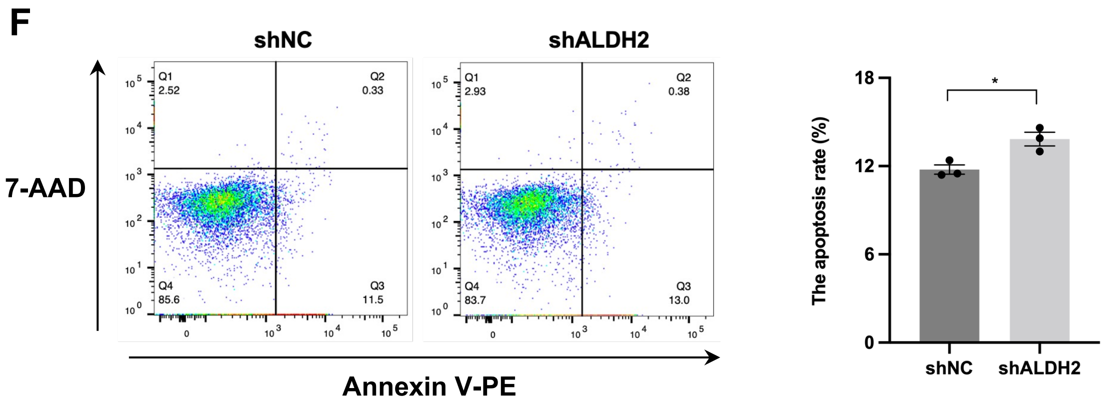

Figure S4 ALDH2 knockdown aggravated the disruption of mitochondria and apoptosis in MA-induced HK-2 cells. HK-2 cells were transfected with ALDH2 shRNA (shALDH2) or negative control shRNA (shNC) plasmid for 6h. (A) The expression of mitochondria-related proteins (ALDH2, PGC-1α and ATP5a1) was measured by western blotting (n=3). (B) The expression of aerobic glycolysis-related enzymes (HK2, PFKFB3 and PKM2) was measured by western blotting (n=3). (C) Measurement of mitochondrial oxygen consumption ratio (OCR) in HK-2 cells (n=5). (D) Measurement of mitochondrial extra cellular acidification rate (ECAR) in HK-2 cells (n=5). (E) The expression of apoptosis-related proteins (Bax and Bcl-2) was measured by western blotting (n=3). (F) Apoptosis was determined by flow cytometry in 2 groups (n=3). $^{*}P <$ 0.05, ** $P < 0.01$, $^{***}P < 0.001$. (shNC, negative control shRNA; shALDH2, ALDH2 shRNA)

Figure S5

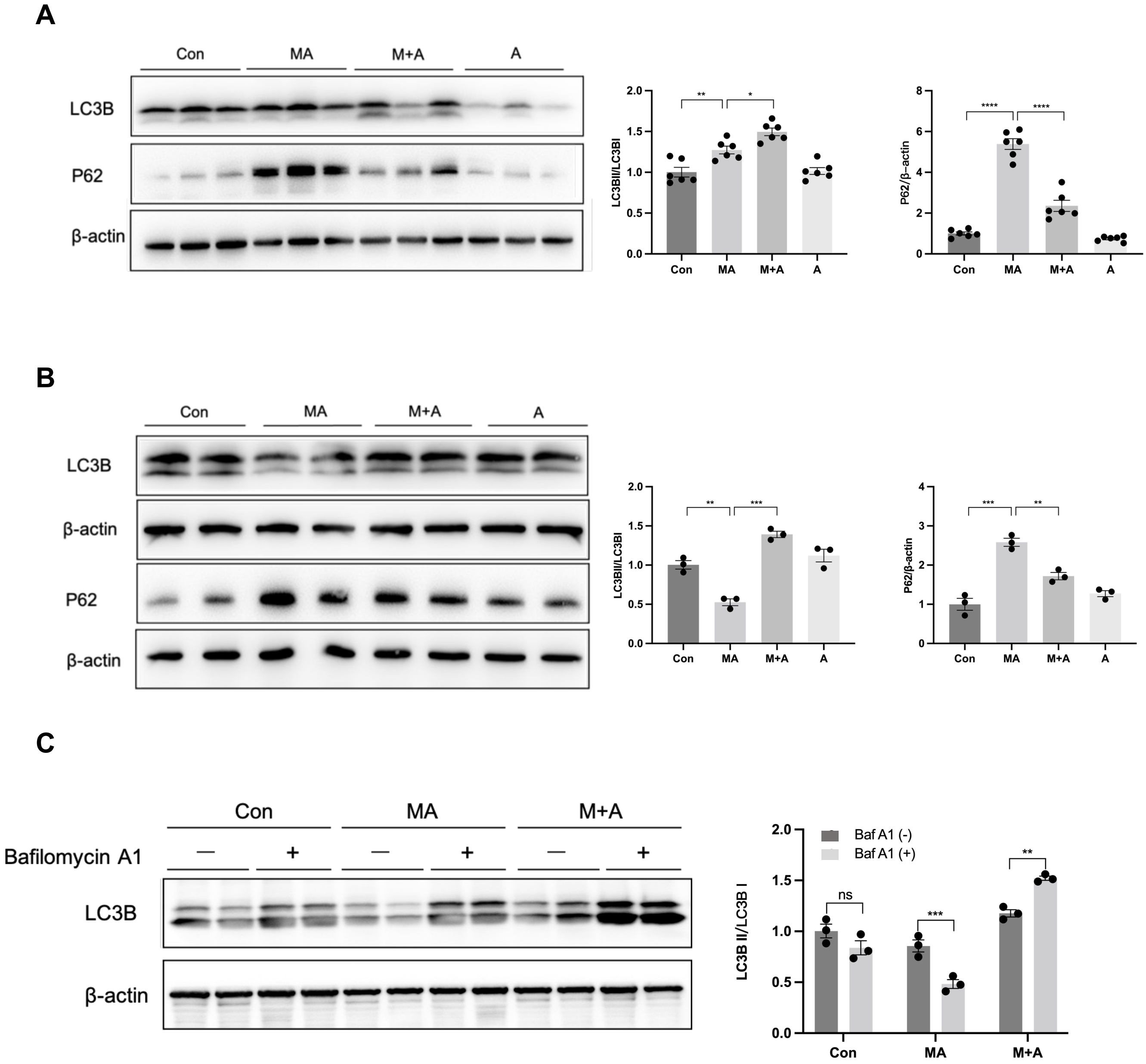

Figure S5 ALDH2 activated autophagy in MA-induced mice and HK-2 cells. (A-B) The expression of autophagy-related proteins (LC3B and P62) was analyzed by western blot in mice (n=6) and HK-2 cells (n=3). (C) The expression of autophagy-related proteins LC3B was analyzed in HK-2 cells stimulated by autophagic inhibitor Baf A1 (n=3). $^{*}P < 0.05$, $^{**}P < 0.01$, $^{***}P < 0.001$, $^{****}P < 0.0001$ (Con, Control; MA, maleic acid; M+A, maleic acid + Alda-1; A, Alda-1; Baf A1, Bafilomycin A1).

Figure S6

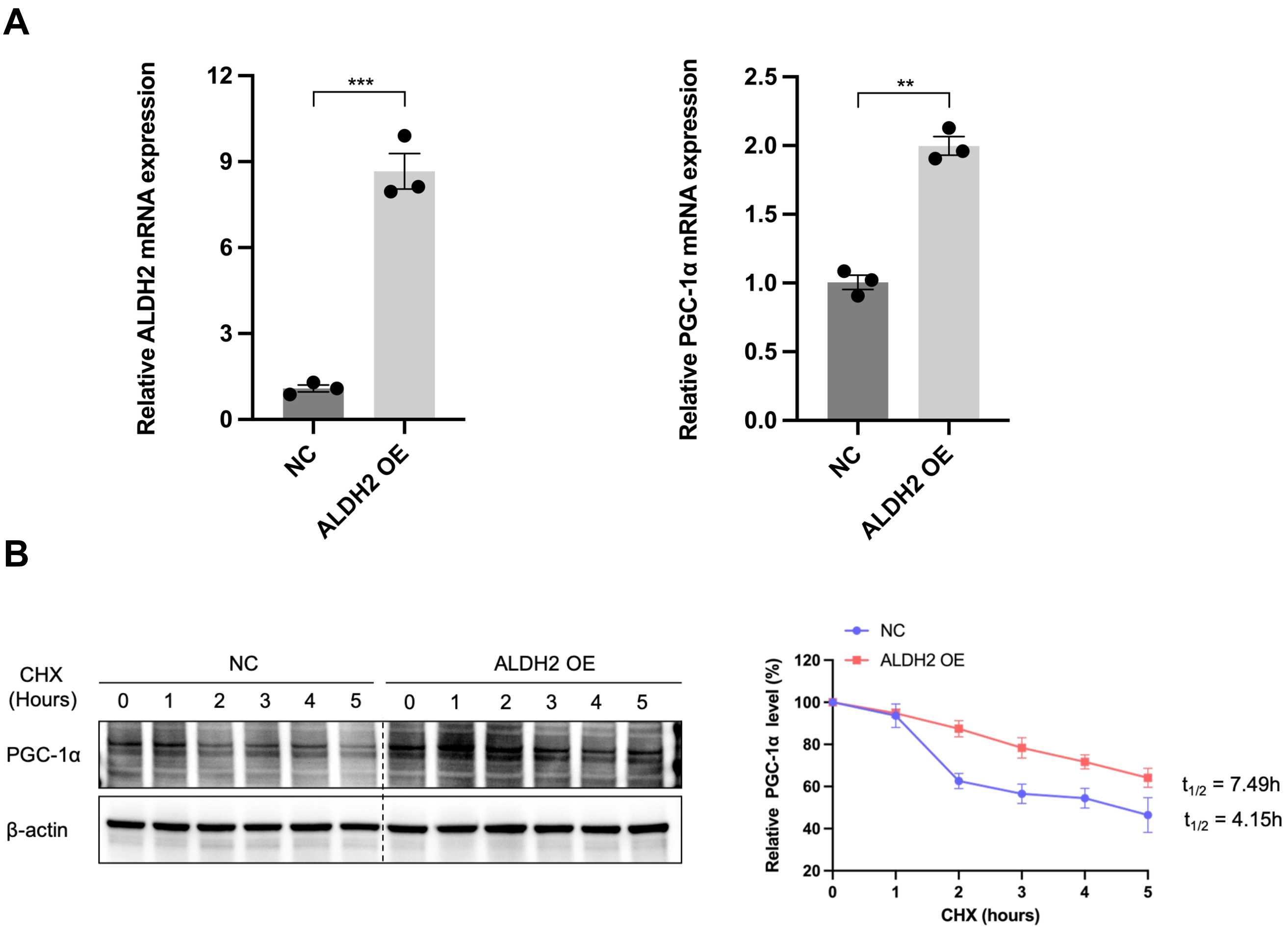

Figure S6 ALDH2 enhanced the expression of PGC-1α by transcriptional and protein half-life regulation. (A) The relative mRNA expression of ALDH2 and PGC-1α was measured in NC and ALDH2 overexpression HK-2 cells (n=3). (B) Cycloheximide (CHX) chase assay for PGC-1α in NC and ALDH2 overexpression HK-2 cells treated with CHX (200 μg/ml) for the indicated time points. $^{*}P < 0.05$, $^{**}P < 0.01$, $^{***}P < 0.001$. (NC, normal control; OE, overexpression; CHX, Cycloheximide)

Table S1 Primers used for real-time PCR analysis.

Gene	Sequence 5’-3’	Species
ALDH2	F: AGAAGATCCTCGGCTACAT<br>R: CTCCTTGGCAATGGTCAT	Mouse
PGC-1α	F: GACACAACACGGACAGAA<br>R: CACAGGTATAACGGTAGGTAA	
mtND1	F: CACACCCTAGCAGAAACAAACC<br>R: GGCCGGCTGCGTATTCTAC	
β-actin	F: CACTGTCGAGTCGCGTCC<br>R: TCATCCATGGCGAACTGGTG	

Table S2 Primary antibodies used in this study.

Antibody	Source	Catalog
ALDH2	Abcam	ab227021
KIM-1	Abcam	ab78494
4HNE	Abcam	ab46545
PGC-1α	ABclonal	A12348
ATP5a1	Proteintech	14676-1-AP
HK2	Abcam	ab209847
PFKFB3	Abcam	ab181861
PKM2	CST	#4053
Bax	ABclonal	A19684
Bcl-2	ABclonal	A19693
SGLT2	ABclonal	A20271
URAT1	Proteintech	14937-1-AP
NPT2a	ABclonal	A13635
Flag	Proteintech	66008-4-Ig
β-actin	ABclonal	AC038
GAPDH	ABclonal	AC033
Histone H3	Proteintech	17168-1-AP
