# Supplementary figures and images for: Aldehyde dehydrogenase 2 alleviates mitochondrial dysfunction by promoting PGC-1α-mediated biogenesis in acute kidney injury
Source: Cell Death Dis. 2023 Jan 20;14(1):45. doi: 10.1038/s41419-023-05557-x (PMC9860042; doi:10.1038/s41419-023-05557-x)

Figure 1

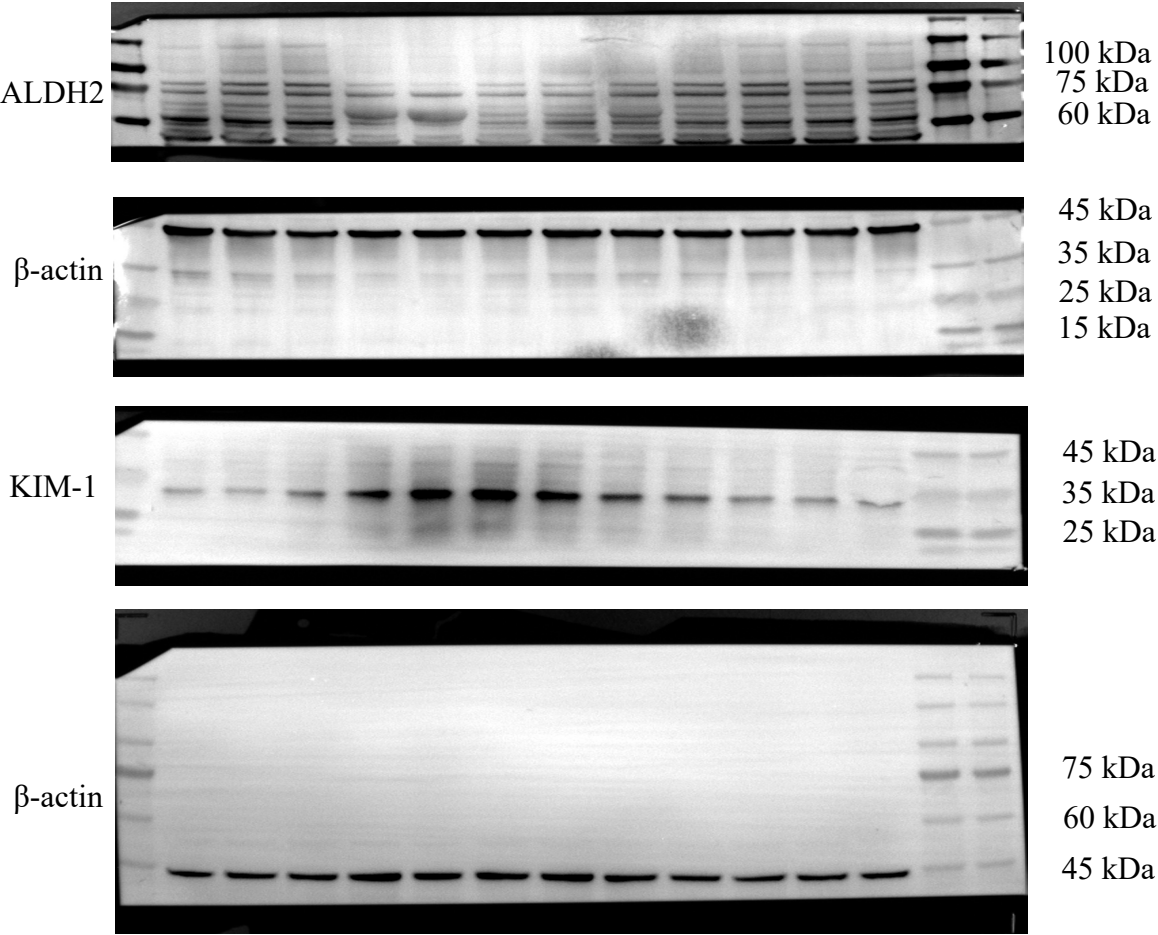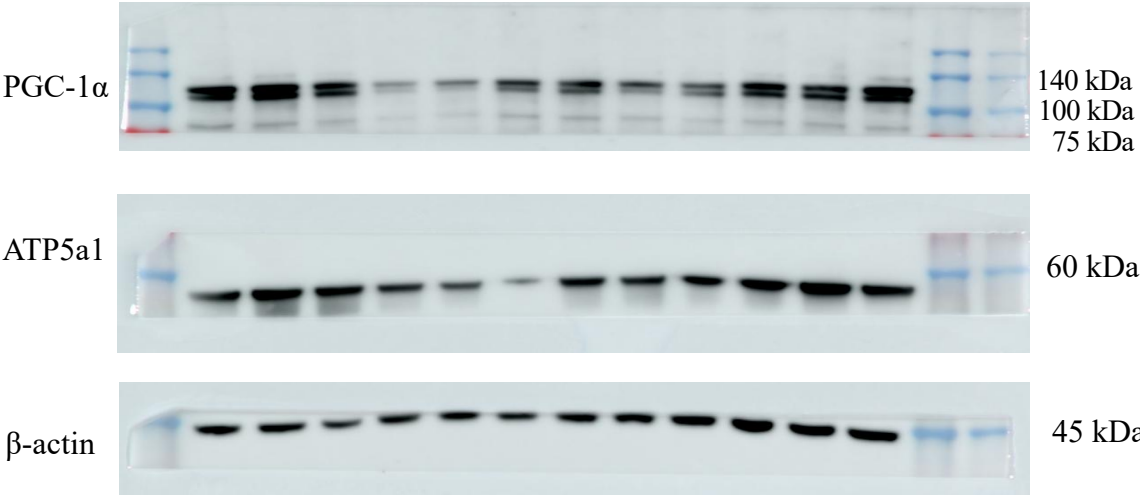

Figure 2

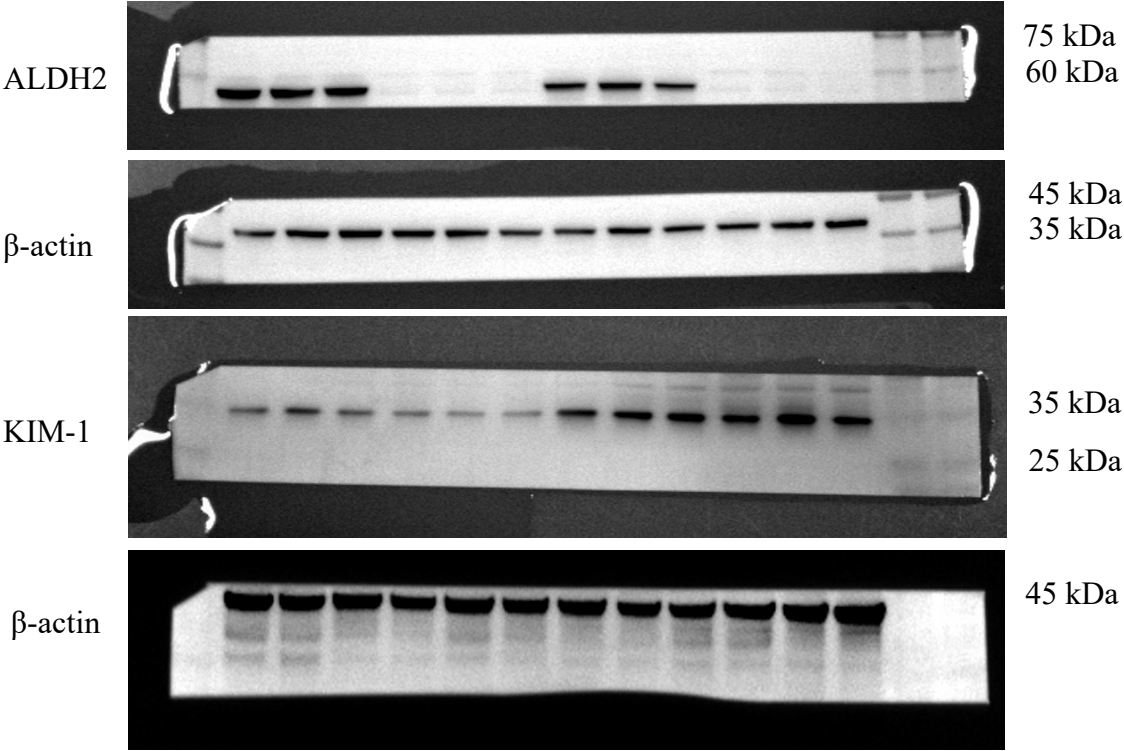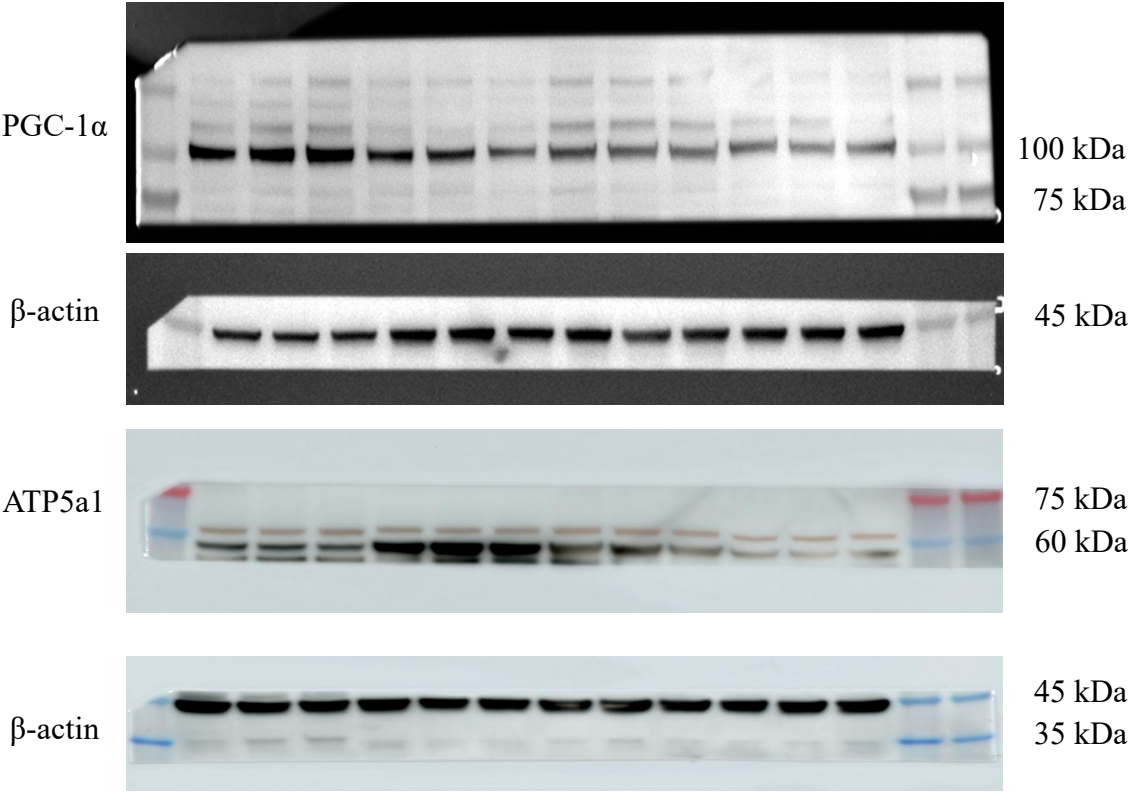

Figure 3

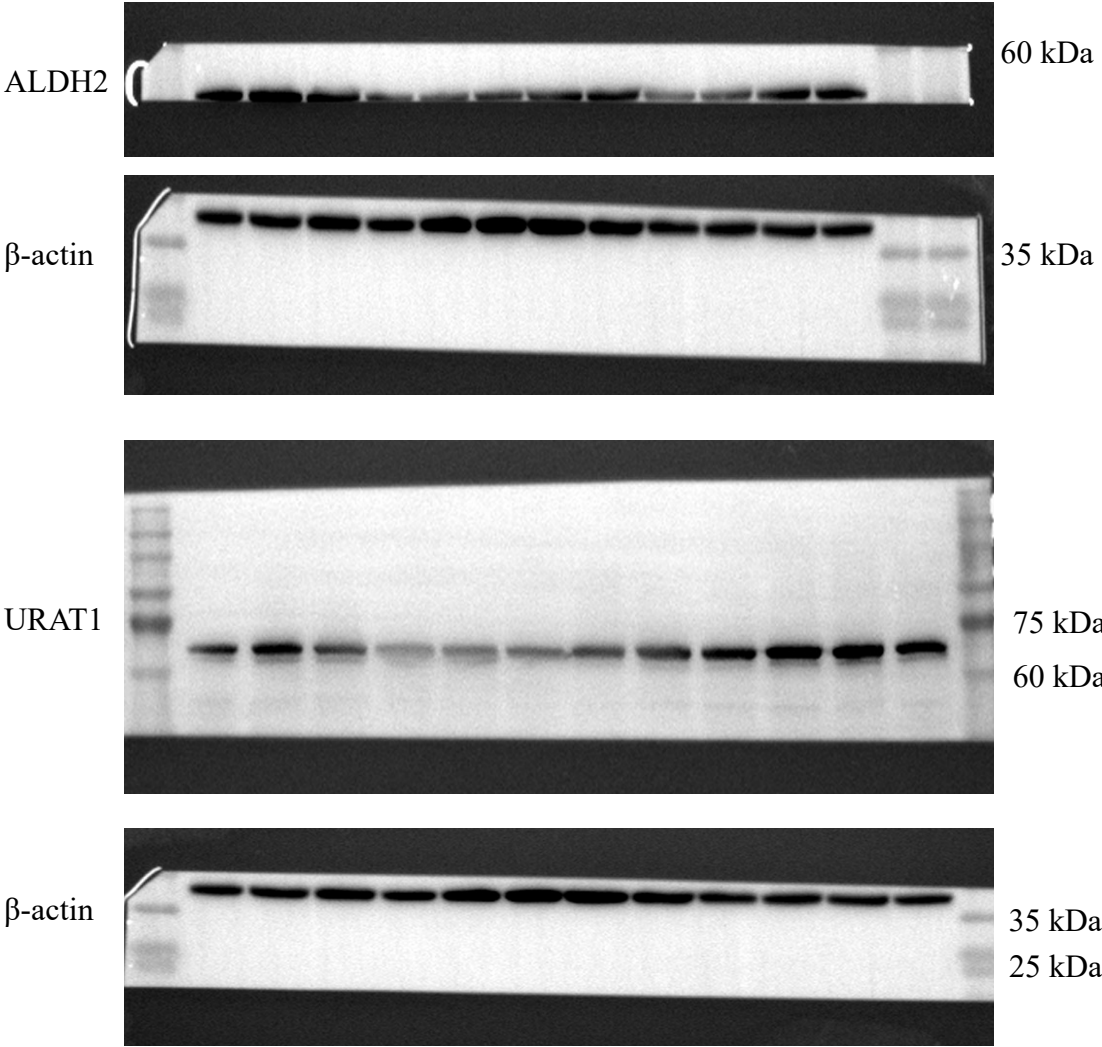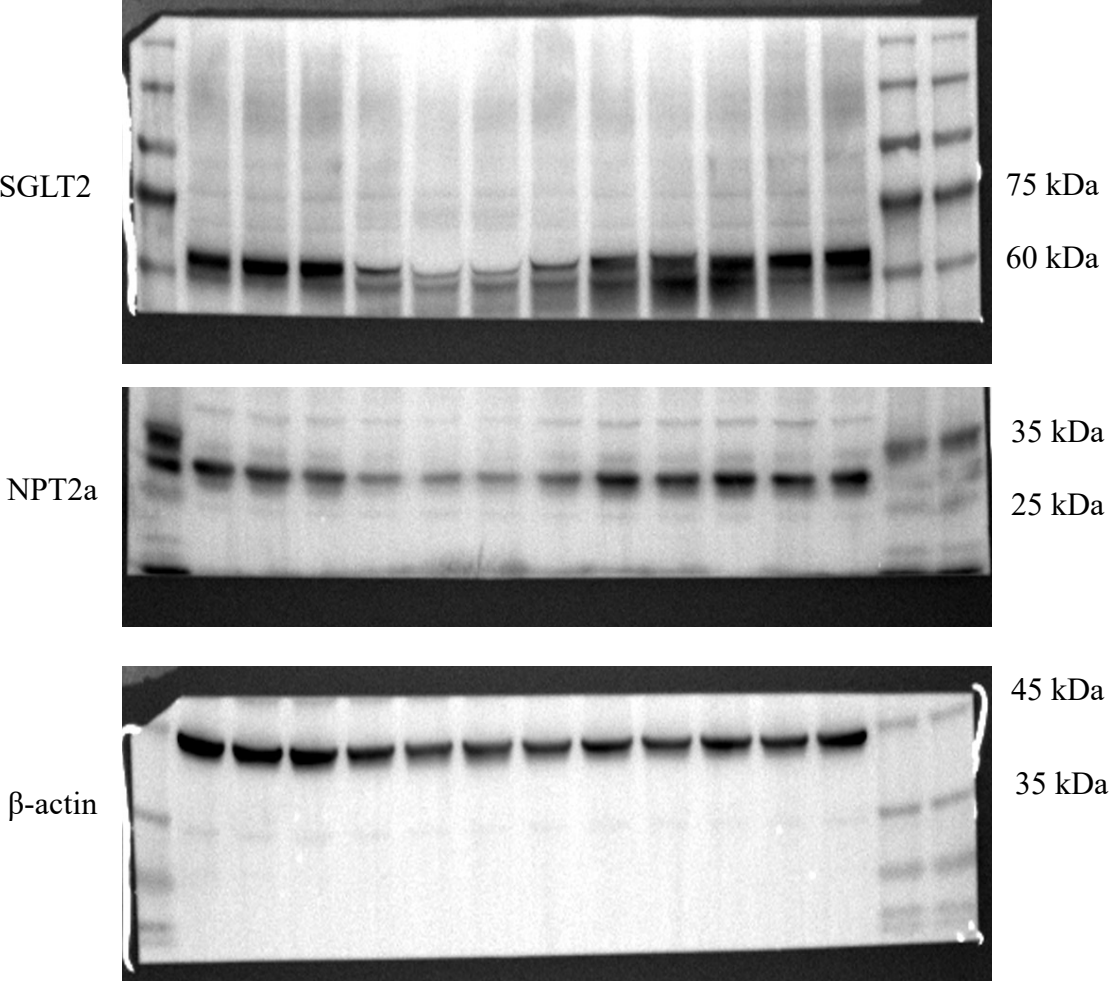

Figure 4

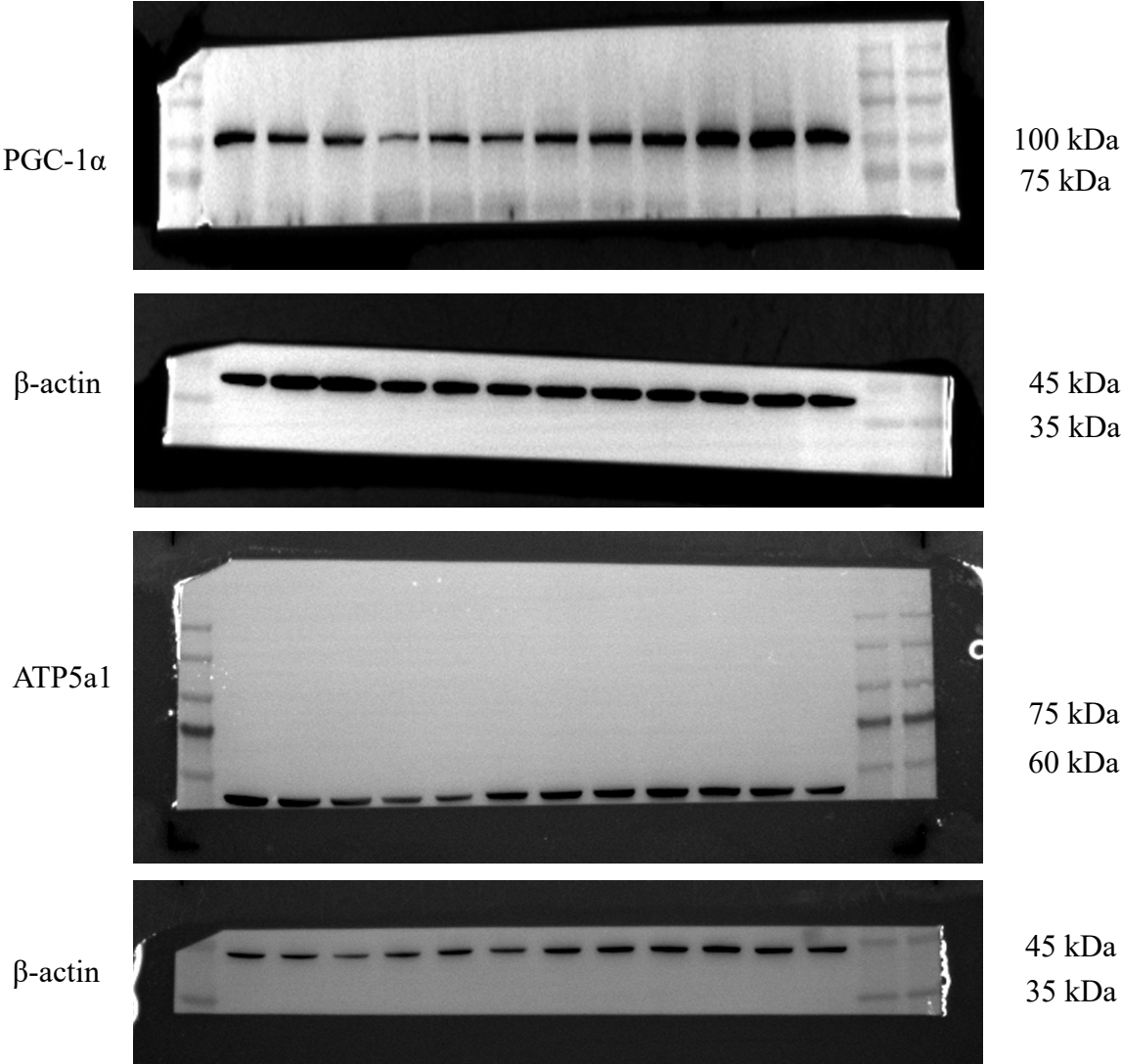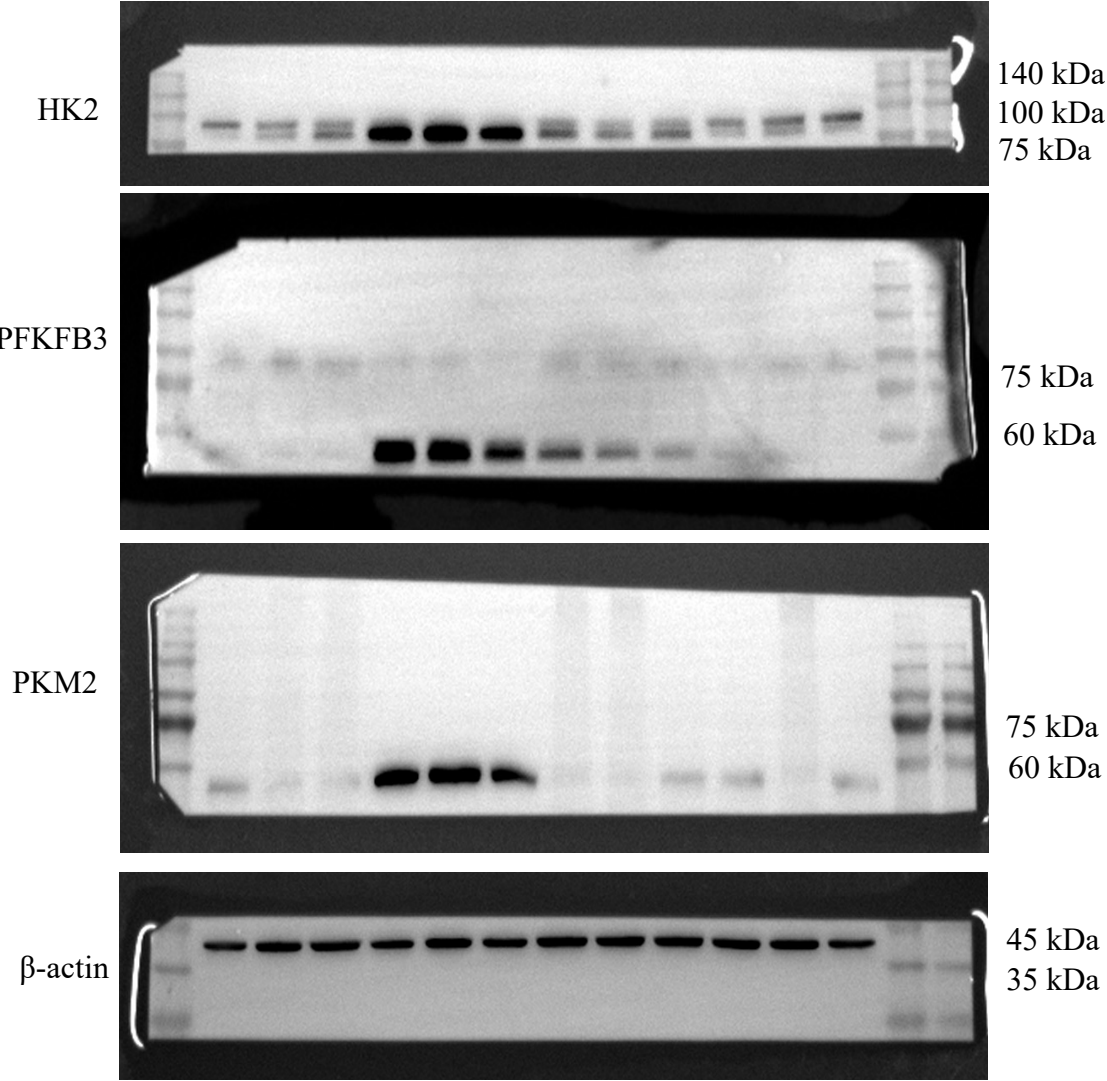

Figure 5

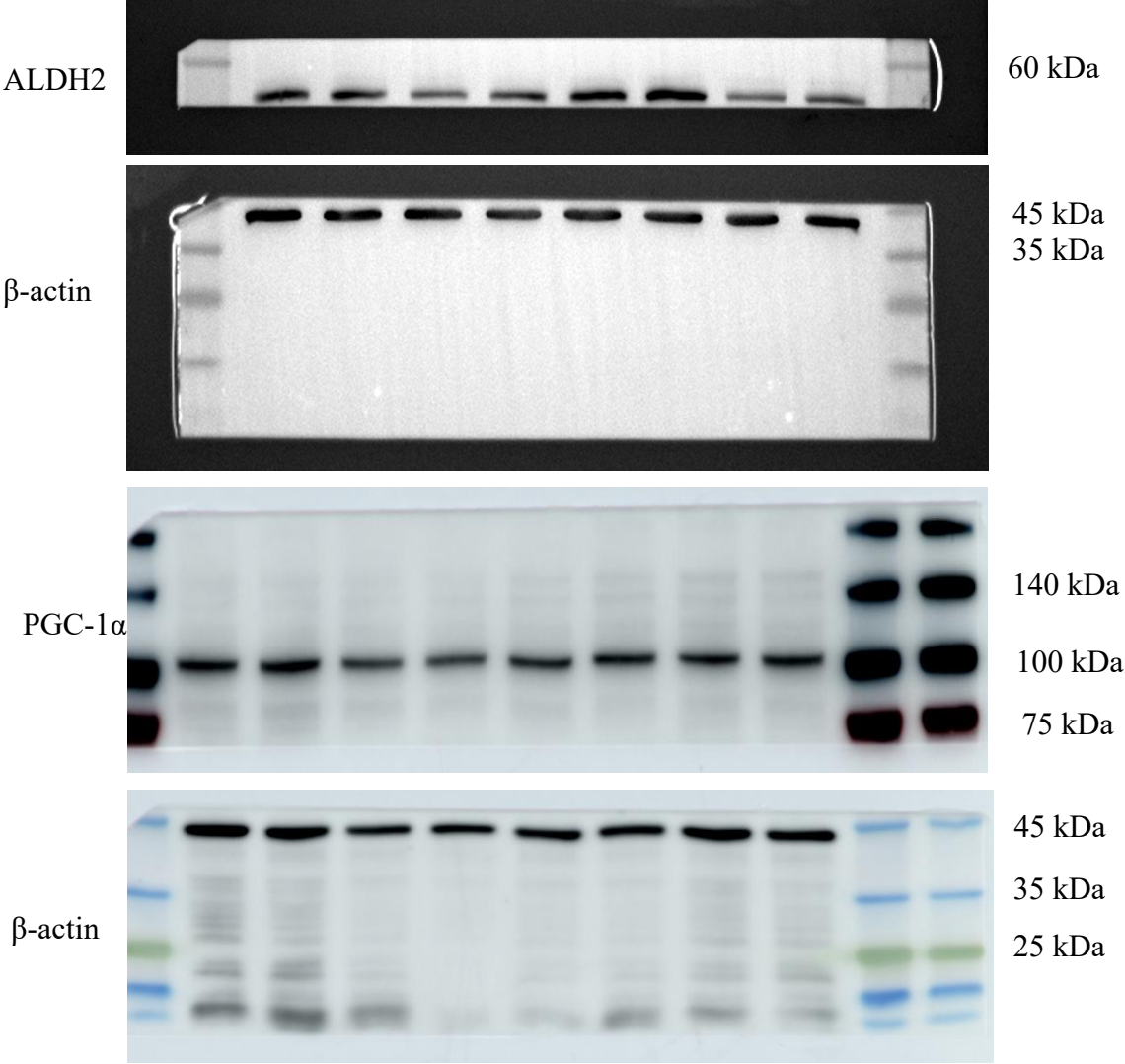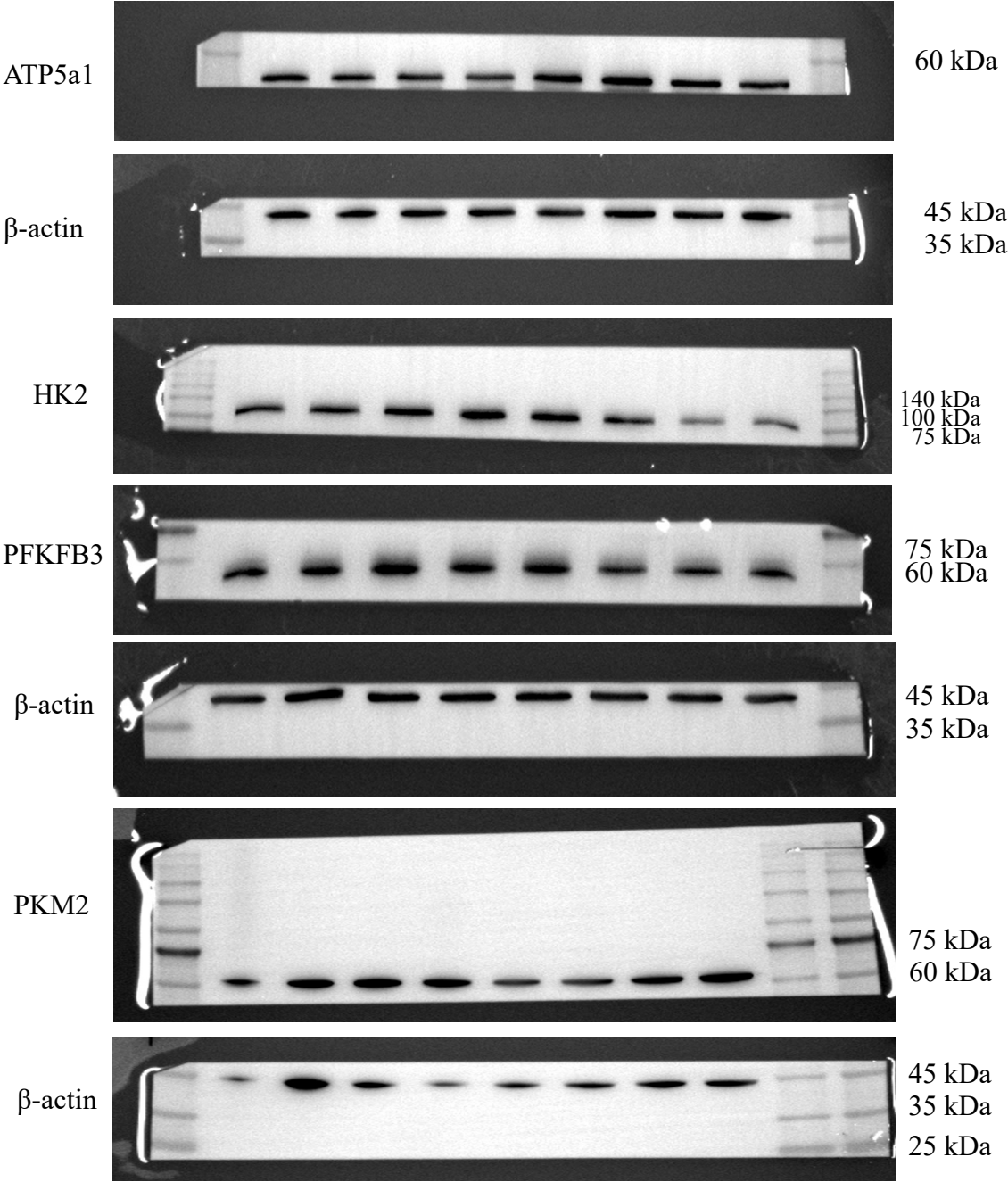

Figure 6

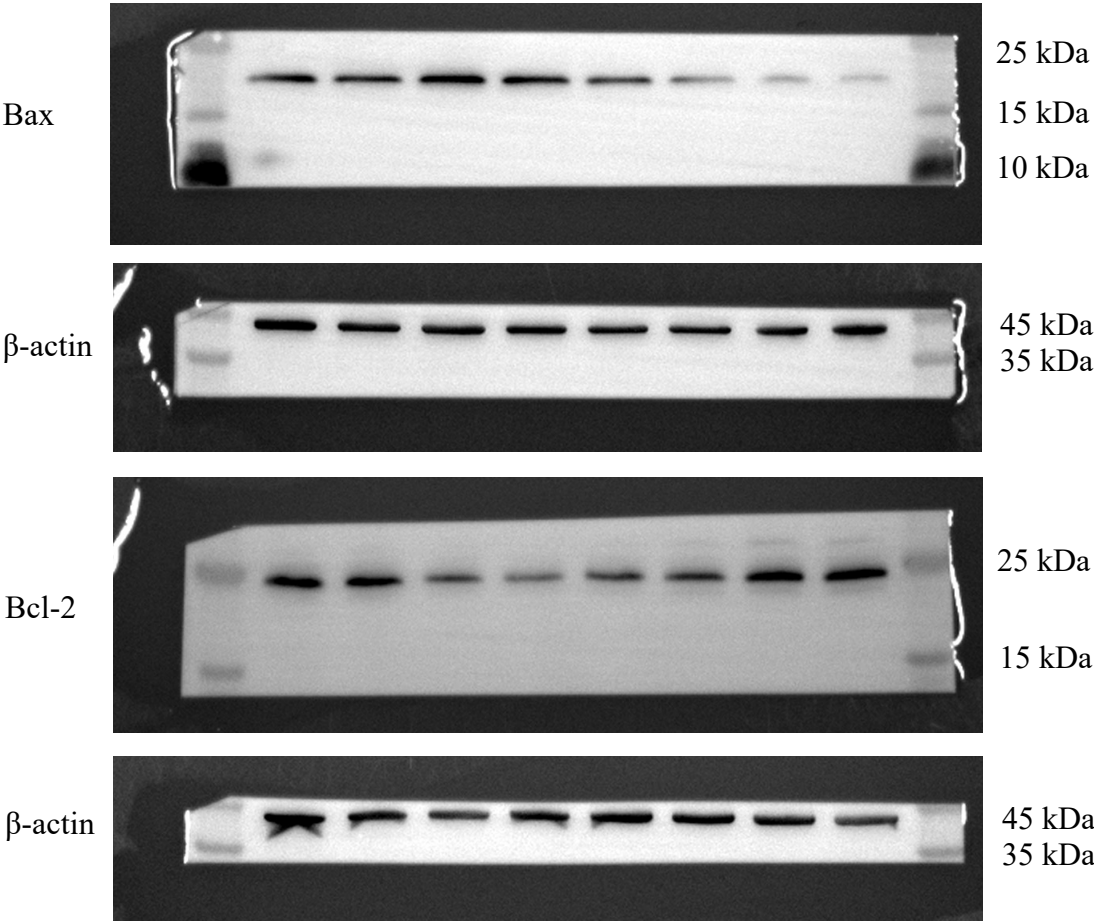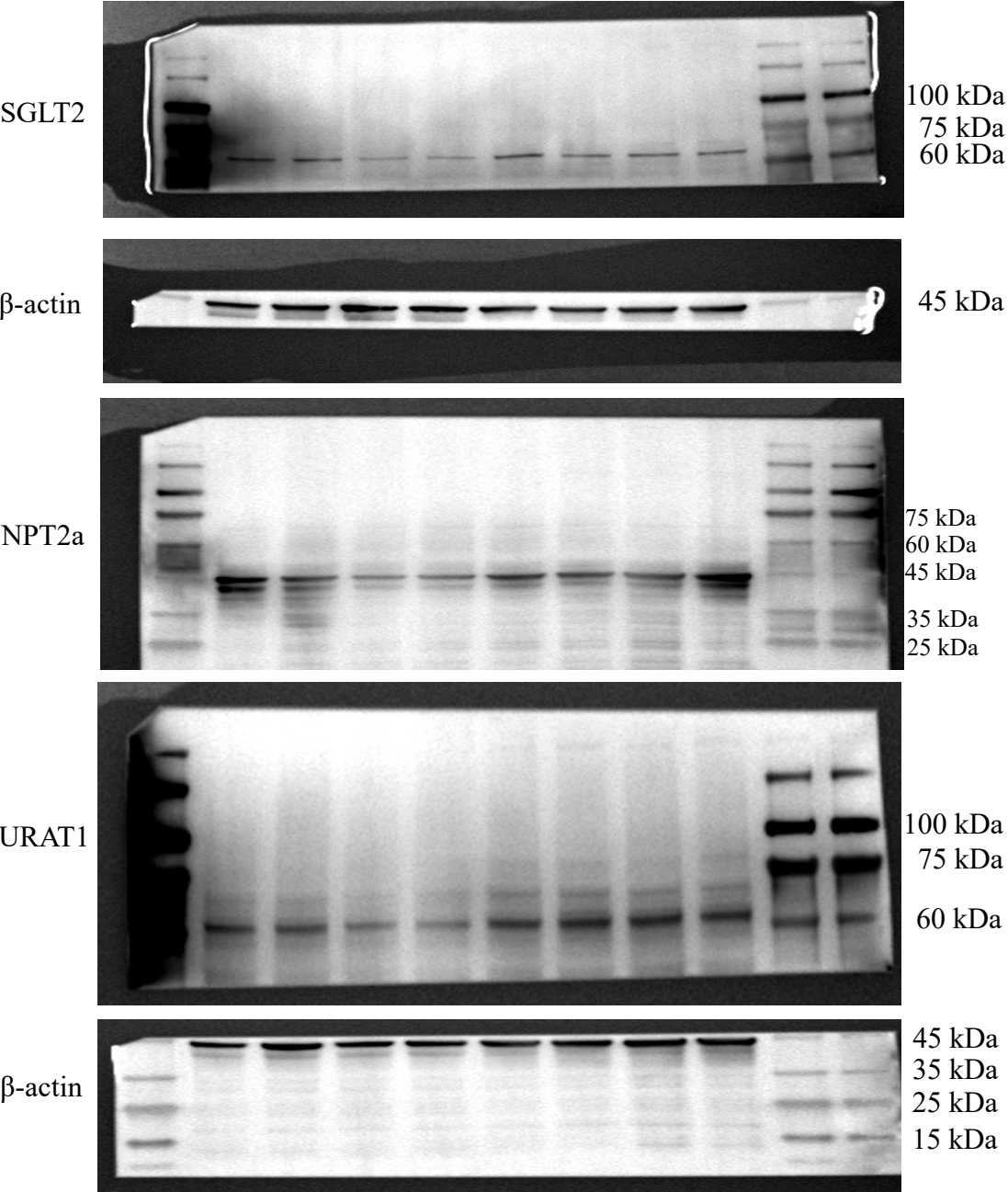

Figure 7B-C

Input

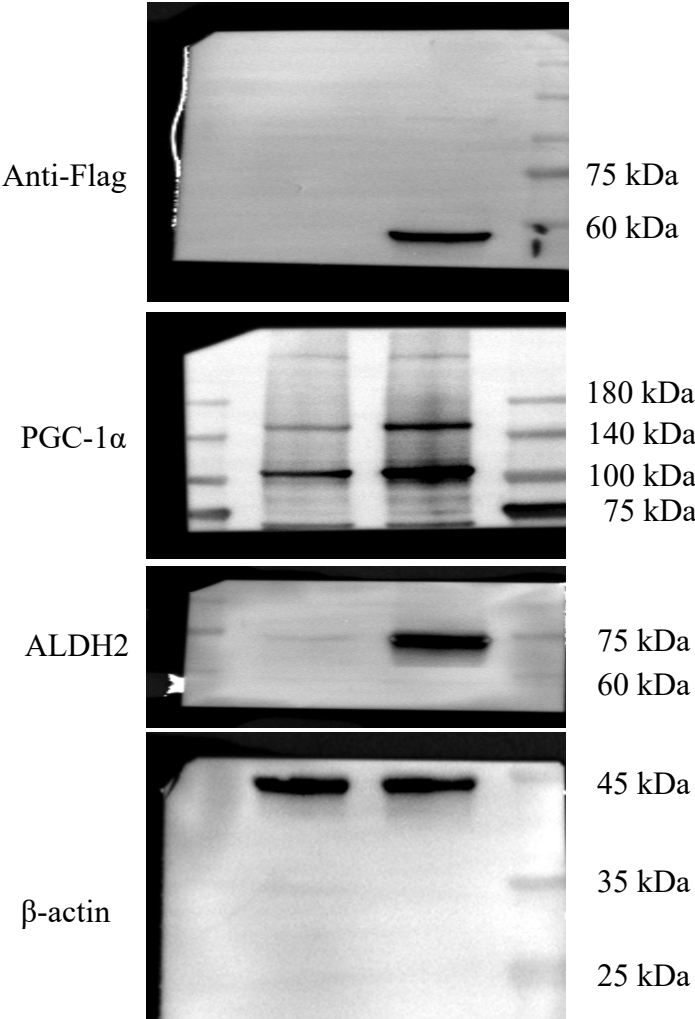

IP

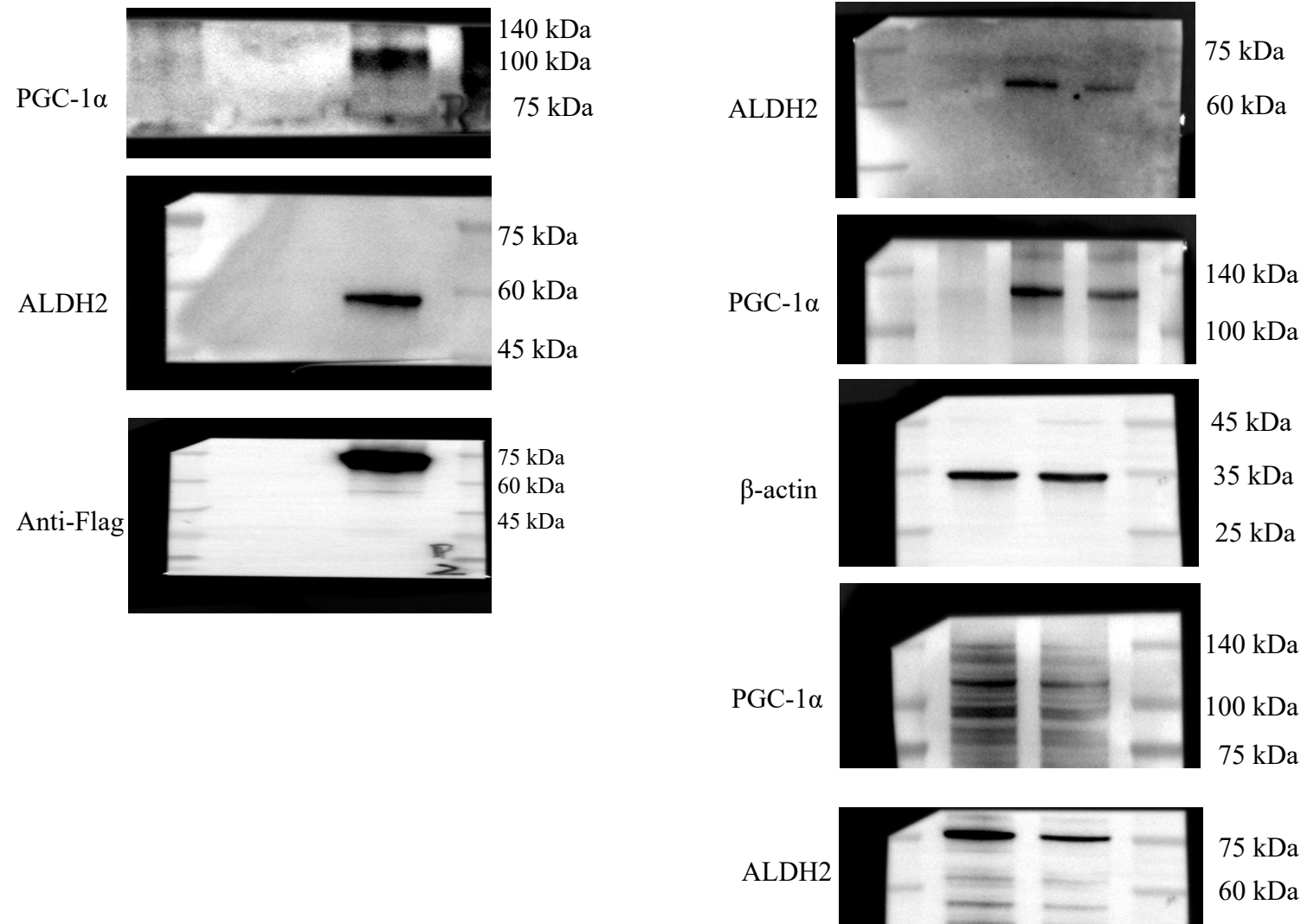

Figure 7D

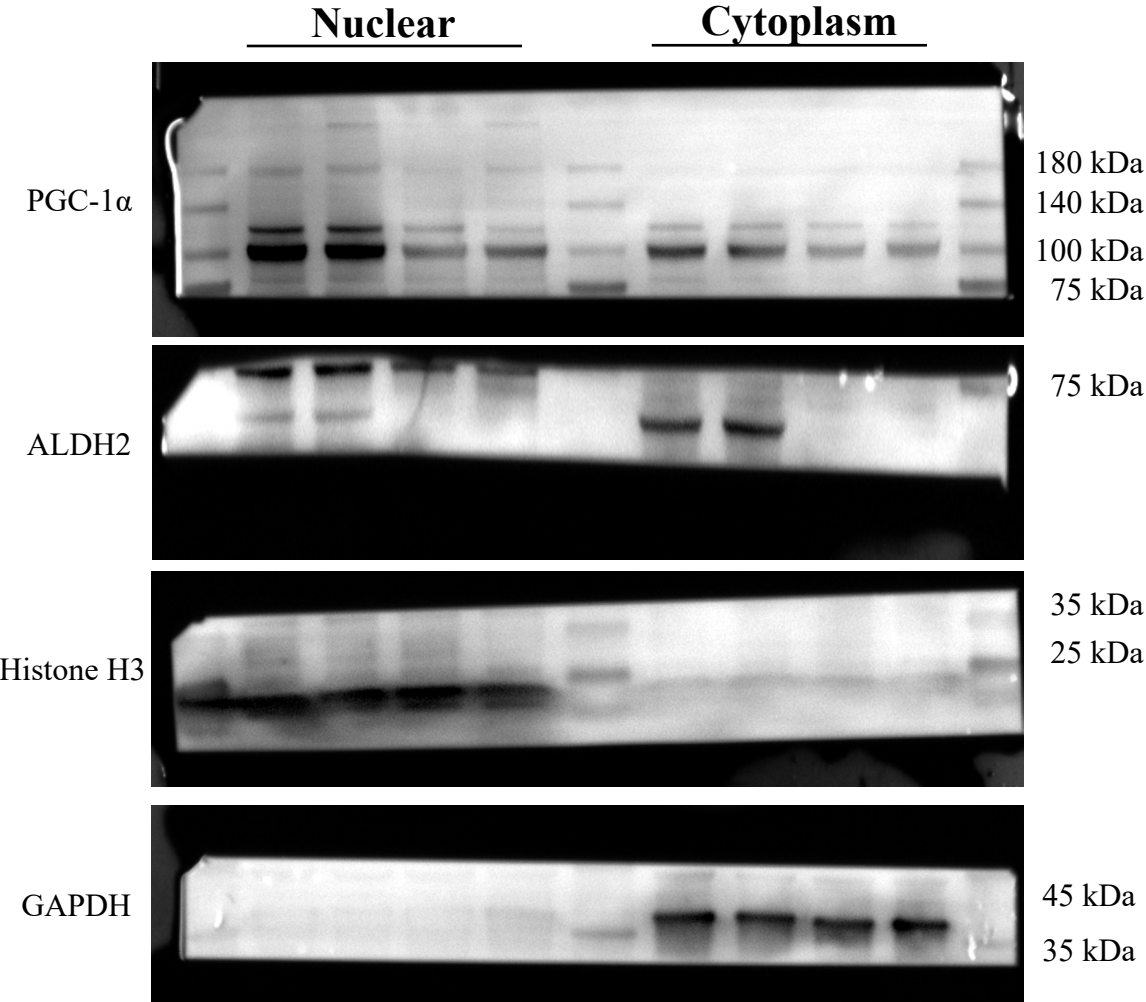

Figure 8

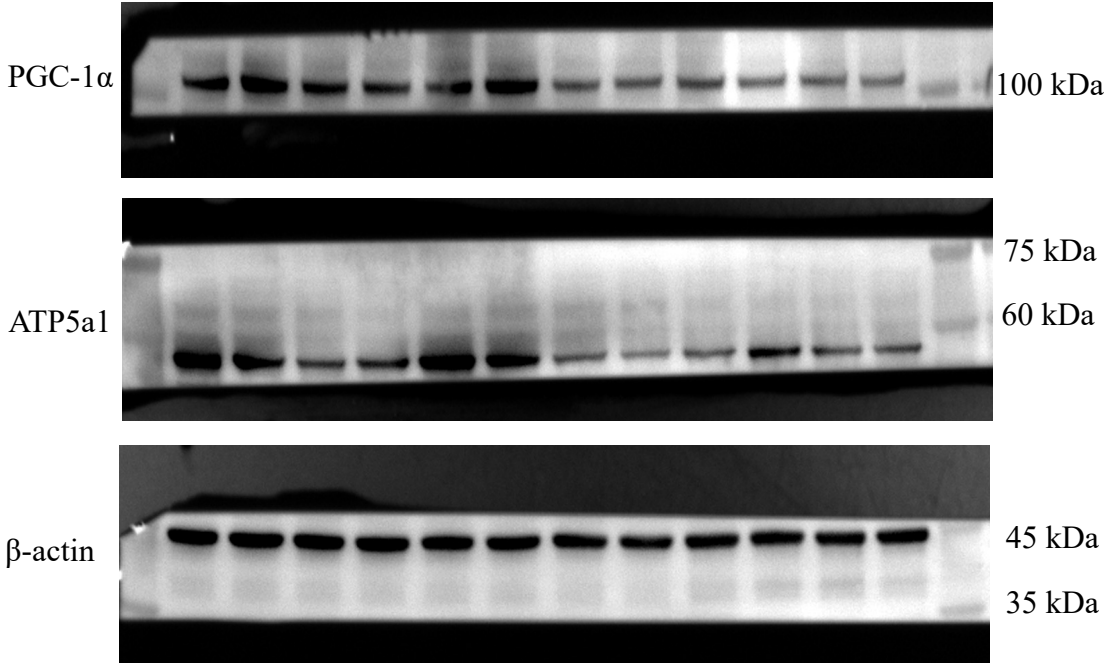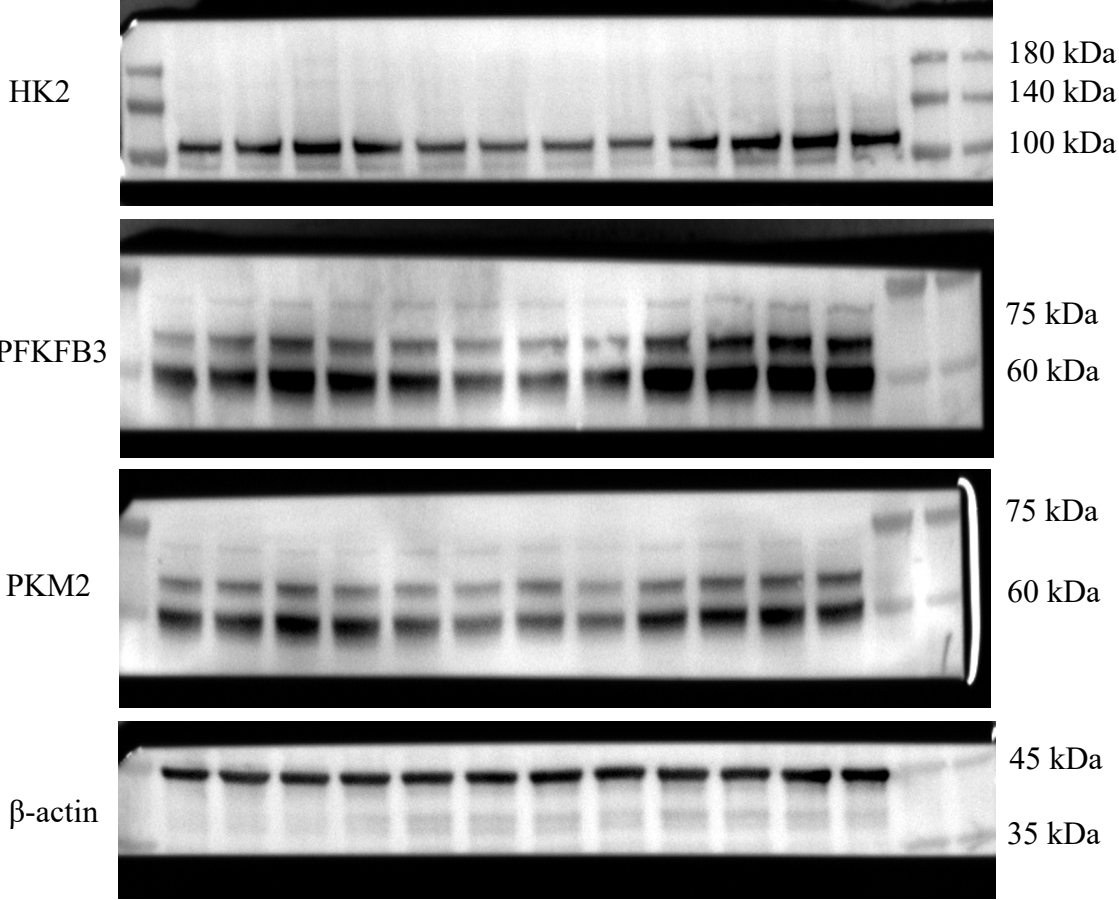

**Figure 8D**

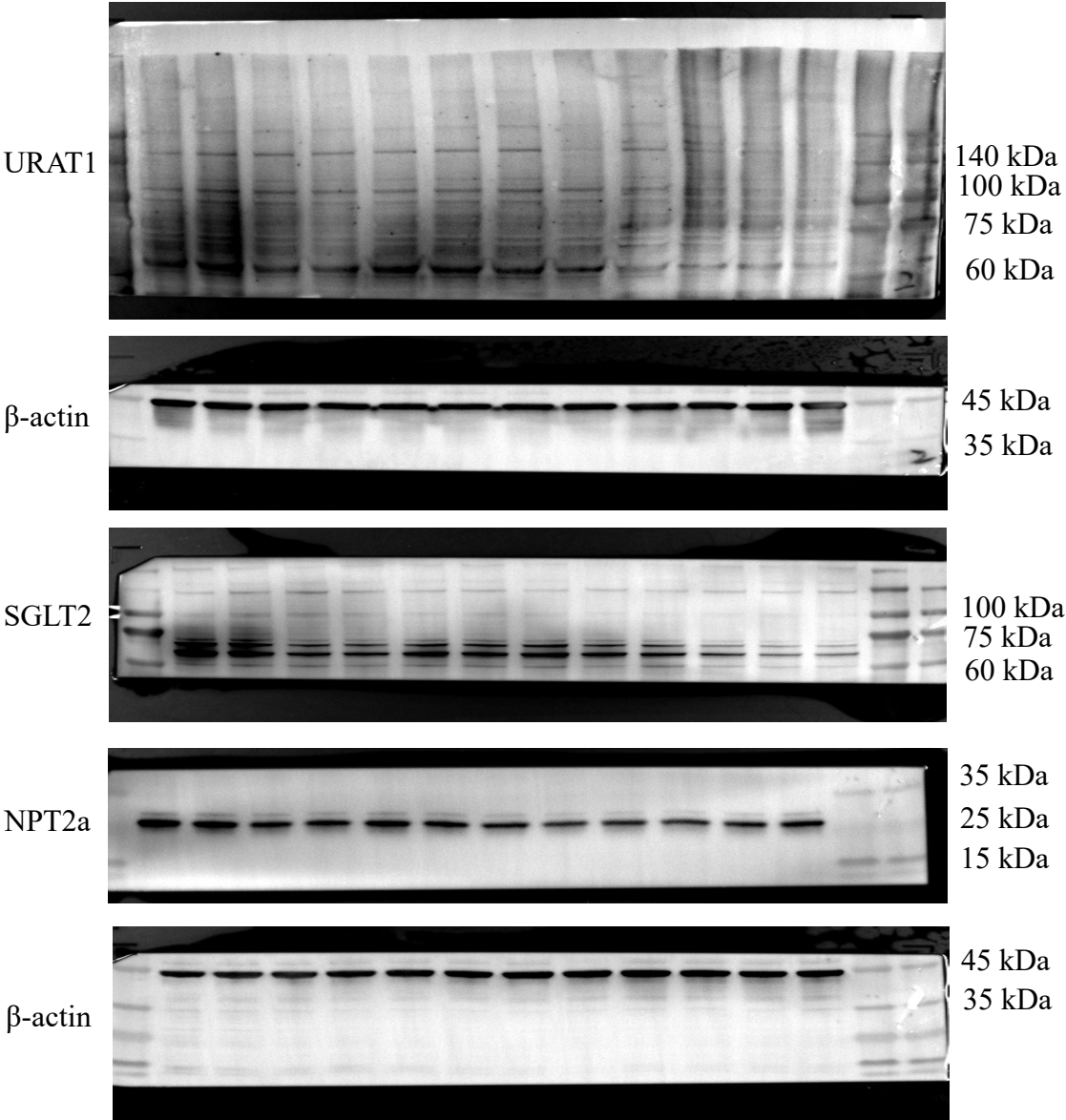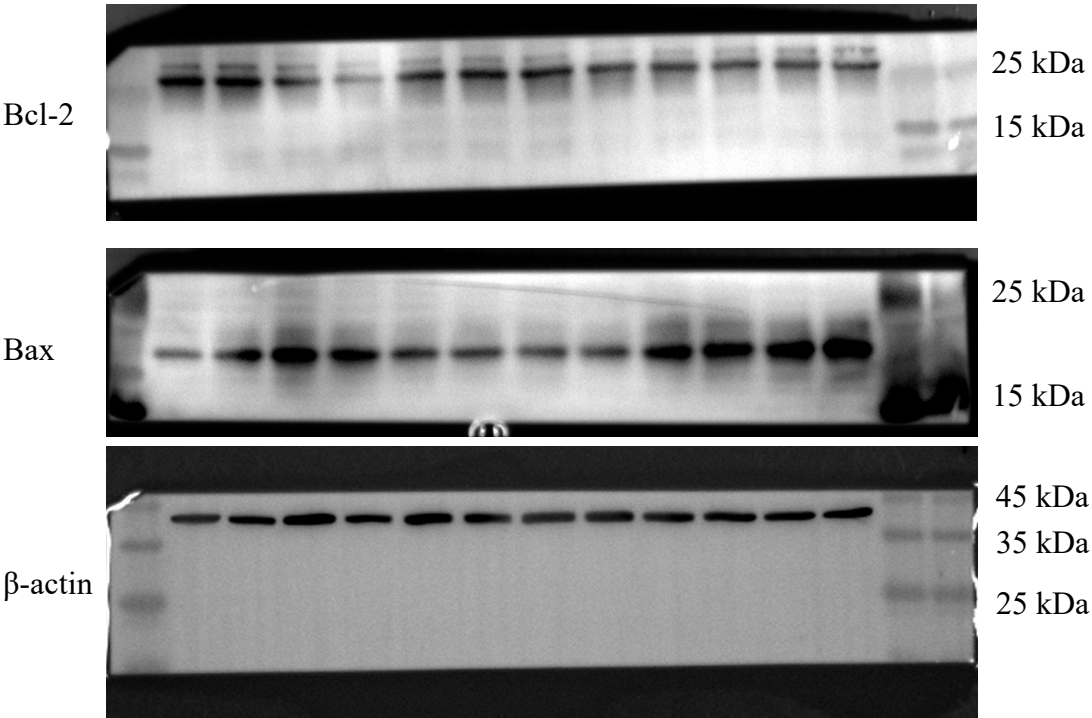

Supplement: Supplementary file 2 — Full length WB [file 41419_2023_5557_MOESM2_ESM.pdf]
